# Optimized metrics for orthogonal combinatorial CRISPR screens

**DOI:** 10.1038/s41598-023-34597-8

**Published:** 2023-05-06

**Authors:** Ronay Cetin, Martin Wegner, Leah Luwisch, Sarada Saud, Tatjana Achmedov, Sebastian Süsser, Antonella Vera-Guapi, Konstantin Müller, Yves Matthess, Eva Quandt, Simone Schaubeck, Chase L. Beisel, Manuel Kaulich

**Affiliations:** 1 Institute of Biochemistry II, Faculty of Medicine, Goethe University Frankfurt, Theodor-Stern-Kai 7, 60590 Frankfurt am Main, Germany; 2 Helmholtz-Centre for Infection Research (HZI), Helmholtz Institute for RNA-Based Infection Research (HIRI), 97080 Würzburg, Germany; 3 Faculty of Medicine and Health Sciences, Universitat Internacional de Catalunya, 08195 Barcelona, Spain; 4 Medical Faculty, University of Würzburg, 97080 Würzburg, Germany; 5 Frankfurt Cancer Institute, 60596 Frankfurt am Main, Germany; 6 Cardio-Pulmonary Institute, 60590 Frankfurt am Main, Germany

**Keywords:** Biological techniques, Genetic engineering, High-throughput screening

## Abstract

CRISPR-based gene perturbation enables unbiased investigations of single and combinatorial genotype-to-phenotype associations. In light of efforts to map combinatorial gene dependencies at scale, choosing an efficient and robust CRISPR-associated (Cas) nuclease is of utmost importance. Even though SpCas9 and AsCas12a are widely used for single, combinatorial, and orthogonal screenings, side-by-side comparisons remain sparse. Here, we systematically compared combinatorial SpCas9, AsCas12a, and CHyMErA in hTERT-immortalized retinal pigment epithelial cells and extracted performance-critical parameters for combinatorial and orthogonal CRISPR screens. Our analyses identified SpCas9 to be superior to enhanced and optimized AsCas12a, with CHyMErA being largely inactive in the tested conditions. Since AsCas12a contains RNA processing activity, we used arrayed dual-gRNAs to improve AsCas12a and CHyMErA applications. While this negatively influenced the effect size range of combinatorial AsCas12a applications, it enhanced the performance of CHyMErA. This improved performance, however, was limited to AsCas12a dual-gRNAs, as SpCas9 gRNAs remained largely inactive. To avoid the use of hybrid gRNAs for orthogonal applications, we engineered the multiplex SpCas9-enAsCas12a approach (multiSPAS) that avoids RNA processing for efficient orthogonal gene editing.

## Introduction

CRISPR screens are a powerful approach to identifying genetic dependencies with single-gene resolution^1–3^. By integrating additional data layers, such as genomic and transcriptional alterations, gene codependencies can be extracted and genetic interactions identified^2,4^. These codependencies are, however, correlative with their biological relevance being limited to sample size and mutagenic spectrum of the additionally used data layers. To accelerate the identification of biologically relevant codependencies, combinatorial CRISPR screens have been developed that can test for direct genetic relationships, map genetic interactions, identify multigenic dependencies, and explore complex biological questions^5–9^.

Currently, two types of combinatorial CRISPR screens are established: single and orthogonal. While single refers to the use of one Cas nuclease, orthogonal refers to the use of different Cas nucleases within the same cell^10^. In contrast to single approaches, orthogonal approaches have the advantage to combine different gene perturbation applications, such as double-strand breaks (knockouts), gene inhibition (CRISPRi), gene activation (CRISPRa), and theoretically enable innovative combinations with base- or prime-editing^11,12^. Several combinatorial screening approaches exist that support single and orthogonal applications^6,8,9,13–18^, with orthogonal approaches being established for SpCas9:SaCas9 (Big Papi) and SpCas9:Lb/AsCas12a (CHyMErA) formats^9,10,19^. However, available data sets from these experiments do not allow side-by-side comparisons and the extraction of performance-critical parameters that are needed to enable the mapping of combinatorial genotype-to-phenotype associations at scale.

While combinatorial CRISPR screens are powerful, they suffer from scalability issues related to large numbers of to-be-investigated query genes^20^. In line with that, we have previously demonstrated that, besides library diversity, library uniformity is a critical size-determining factor of the experimental scale, with uniform libraries (distribution skew; the ratio between the 10th and 90th percentile of normalized read counts is below 2.5) supporting experimental down-scaling and improving scalability and feasibility^13,21^. Despite library formats and their qualitative parameters (e.g. uniformity, completeness), different Cas nucleases and gRNA-expression systems are available for combinatorial screens, with none of them or combinations thereof, yet being robustly established for combinatorial or orthogonal approaches. This is, in part, attributed to the overall lack of quantitative data comparing combinatorial CRISPR technologies^9,19,20^.

In this work, we present a side-by-side and systematic comparison of combinatorial and orthogonal CRISPR screening approaches and identified optimized metrics for combinatorial genotype-to-phenotype associations. A comparison of single and orthogonal SpCas9, AsCas12a, and CHyMErA revealed SpCas9 to perform most robustly. We demonstrate that the need for RNA processing of AsCas12a gRNAs negatively impacts on induction time and strength of gRNA-associated phenotypes. Moreover, we provide evidence that orthogonal CRISPR screens perform optimally with SpCas9 and RNA processing-free enAsCas12a gRNAs. Combining this knowledge, we engineered an orthogonal CRISPR screening approach that generates highly active gRNAs for SpCas9 and enAsCas12a and enables robust and efficient orthogonal genetic perturbations.

## Results

### SpCas9 performs better than enAsCas12a and CHyMErA in combinatorial CRISPR screens

**Figure 1:**
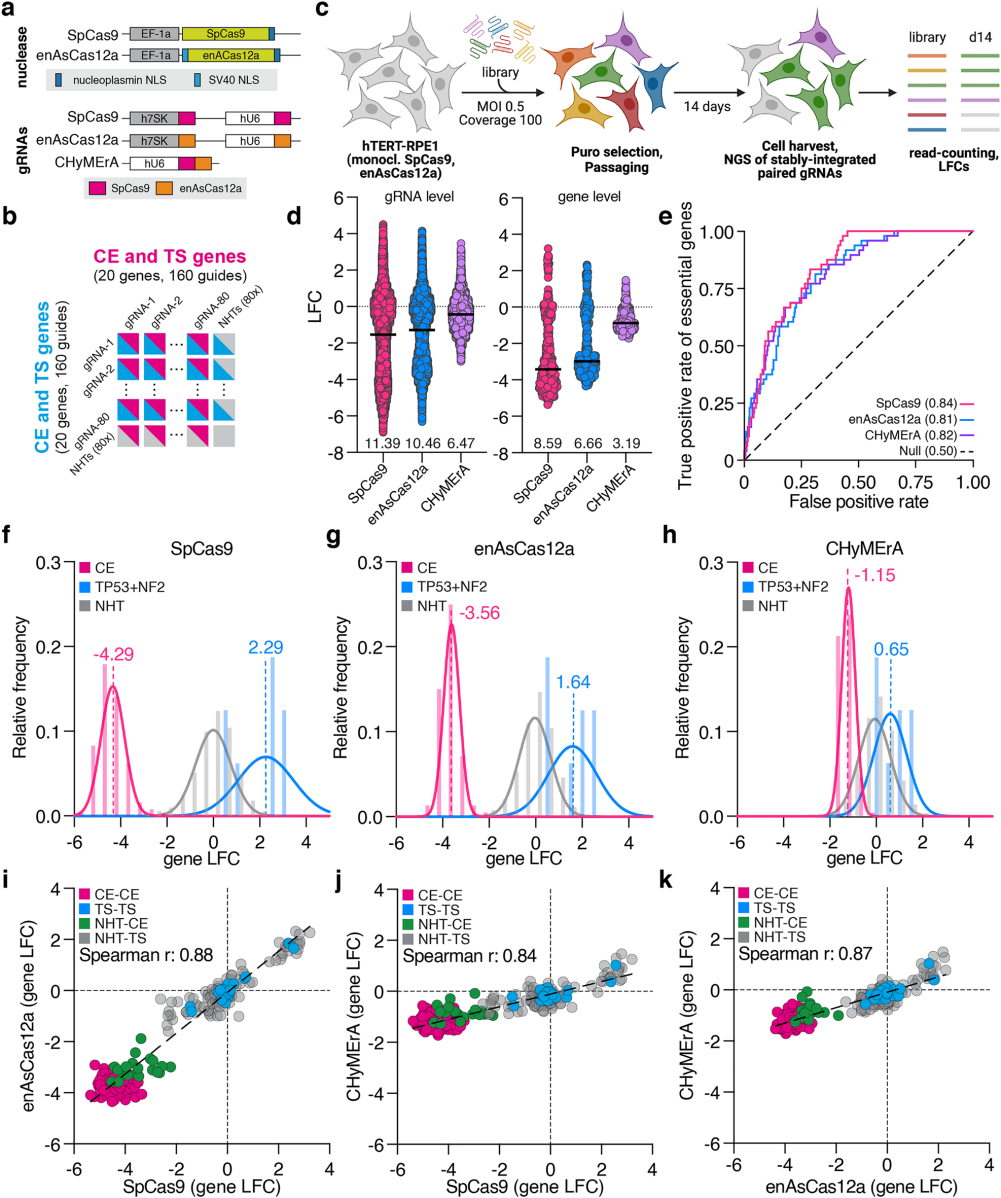
SpCas9 performs better than enAsCas12a in single and combinatorial CRISPR screens. (**a**) Schematic representation of SpCas9 and enAsCas12a nuclease expression constructs and SpCas9, enAsCas12a, CHyMErA gRNA expression constructs. (**b**) Core essential (CE) and tumor suppressor (TS) genes multiplex library design. Combinatorial gRNAs targeting 10 CE and 10 TS genes with 25,600 gRNA combinations; 4 gRNA for each gene together with 80 non-human targeting (NHT) control gRNAs in each cassette. (**c**) CE/TS library screening setup. RPE1 cells were screened with 100-fold library coverage for all three libraries in duplicates. Cells were transduced with an MOI of 0.5. gDNA was harvested on day 14 and prepared for sequencing. End time point and library read counts were used to compute log_2_ fold change (LFC) values. (**d**) LFC values for gRNA pairs and gene pairs for each of the three screens were compared. Black lines represent the median value. The values (effect size range) indicate the difference between the highest and the lowest LFC values. (**e**) Receiver operating characteristic (ROC) curves of gRNAs targeting CE genes for SpCas9, enAsCas12a and CHyMErA screens. The area under the ROC curve (AU-ROC) values are given in parentheses. (**f**) Comparison of library performances. Density plots showing the separation of LFCs of gRNAs targeting CE (pink), NHT (gray), *TP53,* and *NF2* (blue) for SpCas9; and for enAsCas12a in (**g**), and CHyMErA in (**h**). Numbers represent the median LFC values of the population. The solid lines represent the nonlinear fit of the histogram and the bars represent the raw distribution. (**i**) Scatter plot of the LFC comparing CE-CE, TS-TS, NHT-CE/TS gRNA pairs from SpCas9 and enAsCas12a; SpCas9 and CHyMErA (**j**); enAsCas12a and CHyMErA (**k**). CE-CE pairs targeting two CE genes are labeled with pink, TS-TS pairs targeting two TS genes with blue, NHT-CE and NHT-TS pairs targeting a single gene with green and gray respectively. (**i-k**) The linear regression is shown as a dashed line.

To identify a CRISPR-Cas nuclease that supports the unbiased and comprehensive mapping of combinatorial phenotypes in human cells, we compared combinatorial and orthogonal CRISPR screening technologies. To do so, we generated a monoclonal hTERT-immortalized retinal pigment epithelial (hTERT-RPE1, RPE1) cell line constitutively expressing *Streptococcus pyogenes* Cas9 (SpCas9) and the enhanced version of *Acidaminococcus sp.* Cas12a (enAsCas12a)^14^, in the background of wild-type *TP53*, as the recovery of the expected essential genes, regardless of *TP53* status, is feasible in RPE1 cells^22^ (Fig. 1a and Supplementary Fig. S1a). However, due to their different activities and editing outcomes, varying DNA damage responses are expected for Cas9 and Cas12a. To ensure comparable activity and DNA damage responses of SpCas9 and enAsCas12a, the cellular proliferation of RPE1 cells was monitored upon transduction with sgRNAs targeting *TP53* in the absence and presence of Nutlin-3^23^, a small molecule that blocks the *TP53-MDM2* interaction, causing a *TP53*-dependent cell cycle arrest. Similar cell proliferation rates revealed a comparable SpCas9 and enAsCas12a activity, an observation that was independent of *TP53* being targeted by SpCas9 or enAsCas12a (Supplementary Fig. S1b,c). To minimize experimental variation, this cell line was used for all subsequent combinatorial and orthogonal CRISPR screens, if not otherwise stated.

The targeting of fitness-essential genes has previously been used to quantify the performance of CRISPR libraries and screens^24^. We, therefore, focused on a well-characterized set of 10 core-essential (CE) and 10 tumor suppressor (TS) genes, each being targeted by 4 gRNAs derived from an unbiased deep-learning approach^14,25^. 80 non-human-targeting (NHT) control gRNA sequences were added to generate three combinatorial CRISPR libraries for combinatorial and orthogonal applications: (1) SpCas9-SpCas9 (SpCas9), (2) enAsCas12a-enAsCas12a (enAsCas12a), and (3) SpCas9-enAsCas12a (CHyMErA), each containing a total of 25,600 gRNA combinations (Fig. 1a,b and Supplementary Fig. S2a,b)^13,21^. The libraries were applied to drop-out screens in RPE1 cells for 14 days (multiplicity of infection (MOI) 0.5, 100-fold coverage, two technical replicates) (Fig. 1c), after which the abundance of paired gRNAs was determined by deep-sequencing (Supplementary Fig. S3a). Technical reproducibility was high, with individual replicates correlating well on gRNA and gene counts, and gRNA log2-fold change (LFC)-levels (gRNA: SpCas9 *r* = 0.88, enAsCas12a *r* = 0.95, CHyMErA *r* = 0.97; gene: SpCas9 *r* = 0.95, enAsCas12a *r* = 0.99, CHyMErA *r* = 0.97; LFC: SpCas9 *r* = 0.86, enAsCas12a *r* = 0.93, CHyMErA *r* = 0.94) (Supplementary Fig. S3b–d). A simple measure to estimate the overall screen performance is to compute the difference between cells that, depending on which gRNA is present, have increased or decreased fitness. To do so, we computed the range of LFC-values over all gRNA and gene pairs per library (effect size range) and identified SpCas9 to provide the largest effect size range (gRNAs/genes; SpCas9: 11.39/8.59, enAsCas12a: 10.46/6.66, CHyMErA 6.47/3.19) (Fig. 1d). We then evaluated the performance of these libraries by a receiver operating characteristic (ROC) analysis and computed the respective area under the ROC curve (AU-ROC) values by defining gRNAs targeting CE or TS genes as true positives. While their individual effect size varied, the ROC analysis revealed a similar performance of SpCas9 when compared to enAsCas12a or CHyMErA in identifying CE and TS genes (AUC(CE/TS); SpCas9: 0.84/0.96, enAsCas12a: 0.81/0.95, CHyMErA: 0.82/0.92) (Fig. 1e and Supplementary Fig. S1d). Intrigued by this finding, we computed the mean LFC values of gRNAs targeting either CE or TS (only *NF2* and *TP53*) genes and identified SpCas9 to generate the largest separation of negative and positive cell fitness phenotypes (CE/TS; SpCas9: − 4.29/2.29, enAsCas12a: − 3.56/1.64, CHyMErA: − 1.15/0.65) (Fig. 1f–h). With gRNA performance being consistent among the hU6 and h7SK RNA pol III promoters (Supplementary Fig. S4a), these data collectively identify SpCas9 to outperform enAsCas12a and CHyMErA in the tested combinatorial CRISPR screens. *TP53* ablation induces uncontrolled cell proliferation in RPE1 cells, hence, we validated our screening observation by quantifying the induction of cellular proliferation after targeting *AAVS1* and *TP53* genes with SpCas9 or enAsCas12a. Again, this identified SpCas9 to be superior to enAsCas12a, an effect that was independent of the number of used gRNAs (Supplementary Fig. S4b).

However, a larger effect size range does not mean that the identified phenotypes differ. We, therefore, compared gene-level LFCs across screens which revealed an overall high concordance among them (SpCas9-enAsCas12a: *r* = 0.88, SpCas9-CHyMErA: *r* = 0.84, enAsCas12a-CHyMErA: *r* = 0.87) (Fig. 1i–k), which is consistent with our previous AU-ROC analysis (Fig. 1e and Supplementary Fig. S1d), suggesting effect size range and hit ranks being uncoupled, at least for essential and tumor suppressor phenotypes. Together, these results identify SpCas9 to work more robustly than enAsCas12a and CHyMErA in combinatorial CRISPR screens.

### Hybrid guide RNAs decrease effect size range in CHyMErA applications

The CHyMErA platform has been introduced as a combinatorial orthogonal gene editing approach. Here, a hybrid RNA containing a Cas9 and a Cas12a gRNA is expressed from a single U6 promoter. CHyMErA Cas9 and Cas12a gRNAs are designed by established design rules and the CHyMErA-Net algorithm, respectively, collectively resulting in higher gene depletion than both single Cas9 and Cas12a gRNAs alone^9,25^.

**Figure 2:**
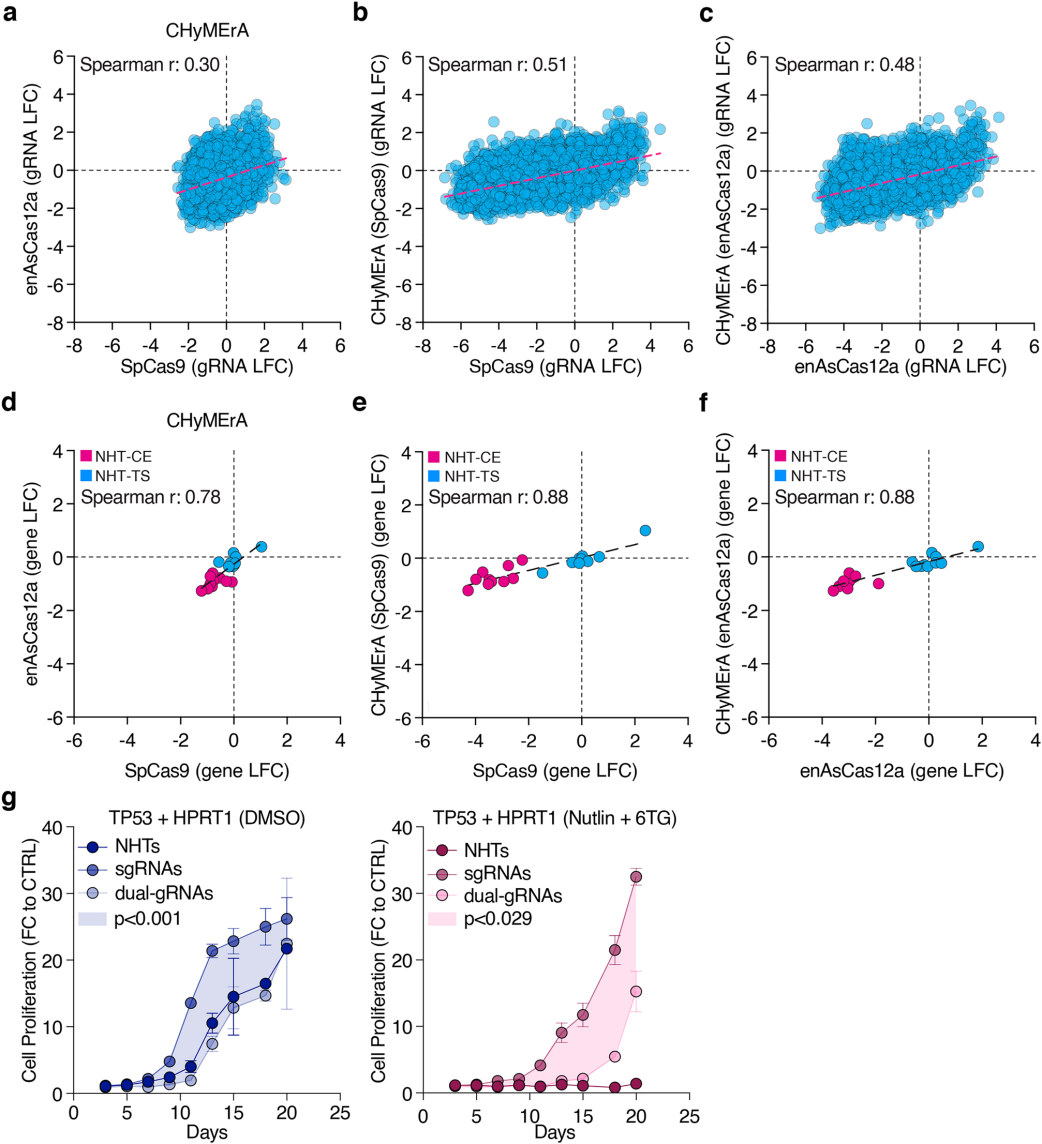
CHyMErA performance is dependent on enAsCas12a gRNA processing activity. (**a**) Comparison of SpCas9 and enAsCas12a gRNA performances from CHyMErA screen. Each data point represents SpCas9 (gRNA-NHT) vs. enAsCas12a (NHT-gRNA) LFC values. (**b**) LFC comparison of SpCas9 gRNAs from SpCas9 and CHyMErA screens, and (**c**) enAsCas12a gRNAs from enAsCas12a and CHyMErA screens. Each data point represents a gRNA expressed from the hU6 promoter. (**d**) Gene level LFC comparison of SpCas9 single gRNAs and enAsCas12a single gRNAs within CHyMErA screens. (**e**) Gene level LFC comparison of SpCas9 single gRNAs within SpCas9 (gRNA:NHT pairs) and CHyMErA (gRNA:NHT pairs) screens. (**f**) Gene level LFC comparison of enAsCas12a single gRNAs within enAsCas12a (gRNA:NHT pairs) and CHyMErA (gRNA:NHT pairs) screens. NHT-CE gRNA pairs targeting a CE gene are highlighted in pink, and NHT-TS gRNA pairs targeting a TS gene in blue. (**g**) Cell proliferation analysis of cells that were transduced with *TP53* and *HPRT1* targeting sgRNAs or dual-gRNAs. Cells were treated either with DMSO (left panel) or Nutlin3 + 6TG (right panel). The shaded area shows the statistical difference between the curves. Data are means of replicates (n = 3). (**d-f**) The linear regression is shown as a dashed line.

Our analysis revealed CHyMErA to greatly underperform compared to SpCas9 and enAsCas12a, not only in effect size range but also in speed of phenotype induction (Figs. 1d,e and 2g). Since CHyMErA gRNAs of the CE/TS library were identical to the gRNAs of the combinatorial SpCas9 and enAsCas12a libraries, we computationally separated CHyMErA hybrid gRNAs into single-transcript SpCas9 or enAsCas12a sgRNAs and compared their performance in combinatorial and orthogonal conditions. Only little concordance could be observed among SpCas9 and enAsCas12a gRNAs within CHyMErA, but also in between CHyMErA and SpCas9 or enAsCas12a libraries (CHyMErA (SpCas9 vs. enAsCas12a): *r* = 0.3, SpCas9 vs. CHyMErA (SpCas9): *r* = 0.51, enAsCas12a vs. CHyMErA (enAsCas12a): *r* = 0.48) (Fig. 2a–c), an effect that only slightly improved when aggregating individual gRNAs to genes (Fig. 2d–f). Ensuring that the observed effect was independent of plasmid design and choice of RNA promoter, we targeted the AAVS1 locus with a single gRNA and determined the editing efficiencies of each plasmid by TIDE^26^. This revealed high and comparable editing rates (> 85%), regardless from which cassette the gRNA was expressed (h7SK or hU6). However, we noticed that the SpCas9 gRNA within the CHyMErA plasmid had lower editing rates (52.7%) when compared to its independent expression (92.2%), as well as the Cas12a gRNA of the CHyMErA plasmid (91.4%) (Supplementary Fig. S10b). CHyMErA gRNAs differ from SpCas9 and enAsCas12a gRNAs in their structure and need for RNA processing^9,27^, we asked if RNA processing, and indirectly their structure, was preventing CHyMErA gRNAs to perform as their unprocessed counterparts. To answer this question, we transduced RPE1 cells with paired gRNA constructs, targeting *TP53* and *HPRT1*, that either required processing (dual-gRNAs) or as single-transcript sgRNAs (sgRNAs). Even in DMSO-treated cells, sgRNAs induced strong and significant cell proliferation, whereas dual-gRNAs were indistinguishable from NHT control guides (Fig. 2g). In line with this, Nutlin-3 and 6TG exposure prevented NHT-transduced cells from proliferating and induced a significantly earlier proliferation of cells transduced with sgRNAs over dual-gRNAs (Fig. 2g). We then performed Northern Blotting to quantify RNA processing of gRNAs derived from the CHyMErA plasmid. This revealed a comparable expression of RNA transcripts and the identification of an unexpected 139 nts-long sequence that could not be detected in the SpCas9 or enAsCas12a samples. This RNA fragment matched the size of the unprocessed SpCas9-enAsCas12a hybrid RNA transcript (Supplementary Fig. S10a). This suggests that, RNA processing is likely not the major cause of the observed differences. However, due to possible improper hybrid RNA folding of CHyMErA gRNAs, it may still account for the slow induction and, ultimately, inefficient separation of gRNA-associated phenotypes.

### Single gene-targeting gRNA arrays compensate for enAsCas12a RNA processing

**Figure 3:**
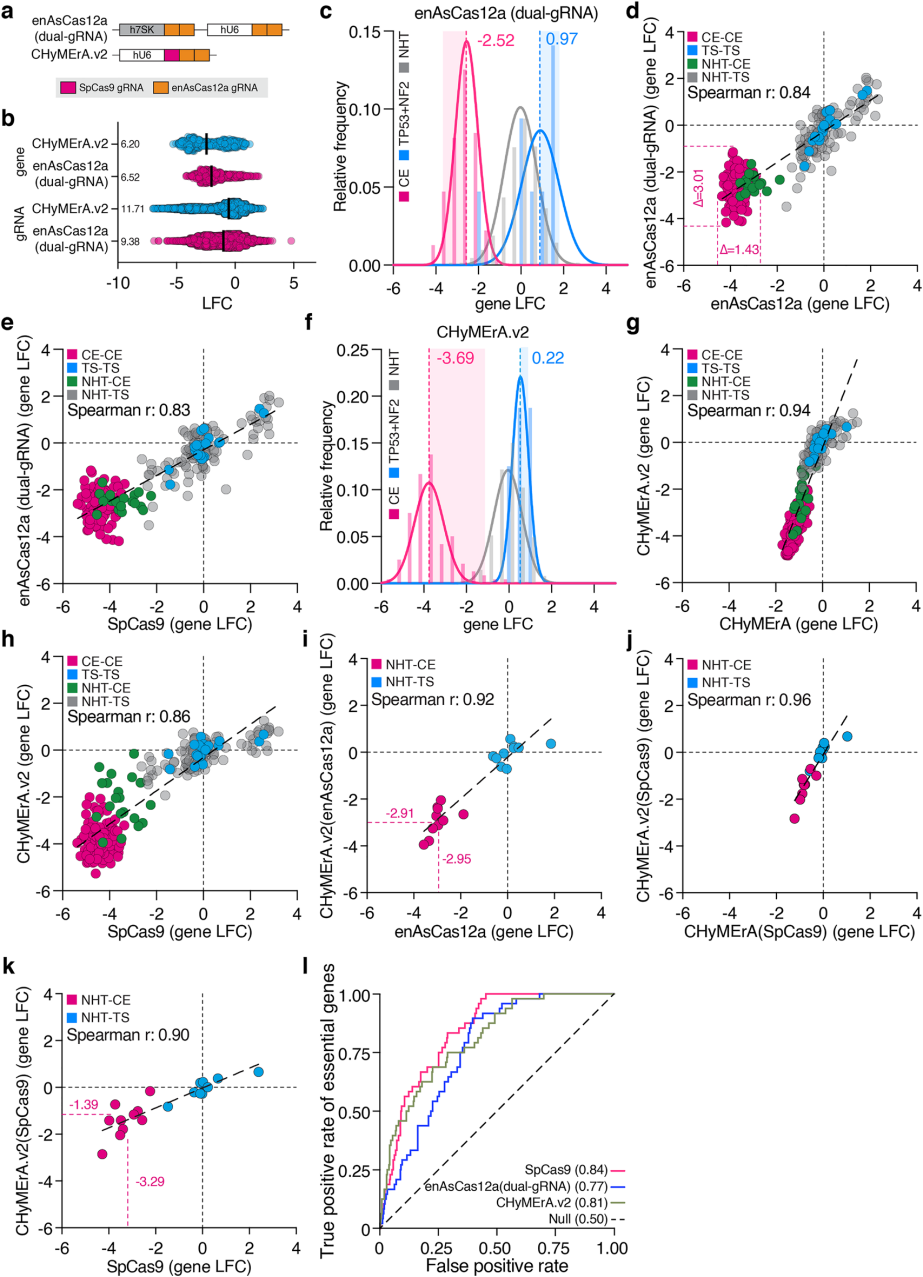
gRNA arrays with single gene targets compensate for RNA processing. (**a**) Schematic representation of enAsCas12a(dual-gRNA) an CHyMErA.v2 gRNA expression constructs. (**b**) Distribution of gene and gRNA level LFC for CHyMErA.v2 (blue) and enAsCas12a(dual-gRNA) (pink). The numbers represent the effect size range of screens by LFC values. Black lines represent the median value. **(c)** Density plot showing the distribution of gene level LFC of enAsCas12a(dual-gRNA), and CHyMErA.v2 in (**f**). CEs are indicated in pink, NHTs are in gray, and *TP53* and NF2 combinations are in blue. Numbers are the median LFC values of the indicated populations. The solid lines represent the nonlinear fit of the histogram and the bars represent the raw distribution. (**d**) Gene level LFC comparison of enAsCas12a and enAsCas12a(dual-gRNA) screens. Delta (Δ) represents the effect size range of CE combinations. (**e**) Comparison of gene level LFCs from SpCas9 and enAsCas12a(dual-gRNA) screens; CHyMErA and CHyMErA.v2 screens (**g**); SpCas9 and CHyMErA.v2 screens (**h**). (**i**) Gene level comparison of enAsCas12a gRNAs from CHyMErA.v2 and enAsCas12a screens. (**j**) Gene level comparison of SpCas9 gRNAs from CHyMErA and CHyMErA.v2 screens. (**k**) Gene level comparison of SpCas9 gRNAs from CHyMErA.v2 and SpCas9 screens. (**l**) Receiver operating characteristic (ROC) curves for guides targeting CE genes for SpCas9, enAsCas12a(dual-gRNA) and CHyMErA.v2 screens. The area under the ROC curve (AU-ROC) values are given in parentheses. (**d,e,g,h**) CE-CE pairs targeting two CE genes are labeled with pink, TS-TS pairs targeting two TS genes with blue, NHT-CE, and NHT-TS pairs targeting a single gene with green and gray respectively. (**i**–**k**) NHT-CE gRNA pairs targeting a CE gene are highlighted in pink, and NHT-TS gRNA pairs targeting a TS gene are in blue. The dashed line is the linear regression line.

The ability of enAsCas12a to process polycistronic gRNAs is a powerful means to improve single-gene knockout rates and enable multi-gene edits^28,29^. Even though RNA processing negatively impacted phenotype induction and effect size range of CHyMErA, we asked if polycistronic gRNA expression would improve the performance of combinatorial enAsCas12a and CHyMErA approaches by targeting two different loci in the same gene with two pre-defined gRNAs. To this end, we designed two additional 3Cs library plasmids enAsCas12a(dual-gRNA) and CHyMErA.v2 (Fig. 3a), and used them to generate combinatorial CRISPR libraries targeting the same set of CE and TS genes as before (Fig. 1b and Supplementary Fig. S5a,b). Screens were performed in the SpCas9 and enAsCas12a-expressing RPE1 cells with paired-end gRNA sequencing after 14 days of culture (MOI 0.5, 100-fold coverage, two technical replicates) (Supplementary Fig. S6a). Again, the replicate correlation was high on gRNA and gene counts, as well as gRNA LFC-levels (gRNA: enAsCas12a(dual-gRNA) *r* = 0.93, CHyMErA.v2 *r* = 0.95; gene: enAsCas12a(dual-gRNA) *r* = 0.91, CHyMErA.v2 *r* = 0.90; gRNA-LFC: enAsCas12a(dual-gRNA) *r* = 0.88, CHyMErA.v2 *r* = 0.91) (Supplementary Fig. S6b–g). Interestingly, gRNA and gene effect size range, as well as the mean LFC of CE and TS genes derived from enAsCas12a(dual-gRNA) screens were decreased compared to single sgRNAs (Fig. 3b,c). Moreover, when analyzing the gene LFC distribution of gRNAs targeting core essential genes, the range over which phenotypes developed more than doubled from 1.43 to 3.01 (Fig. 3d). Despite these changes, gene ranks correlated well among enAsCas12a(dual-gRNA), enAsCas12a and SpCas9 (enAsCas12a(dual-gRNA) vs. enAsCas12a: r = 0.84, enAsCas12a(dual-gRNA) vs. SpCas9: r = 0.83)) (Fig. 3d,e). Together, these data demonstrate that gRNA processing not only negatively impacts the effect size range of enAsCas12a CRISPR screens but also reduces the derived confidence in phenotypes.

Next, we investigated the effect of arrayed enAsCas12a gRNAs on the performance of CHyMErA.v2. In contrast to enAsCas12a(dual-gRNA), we observed a markedly improved performance when compared to the original CHyMErA (Fig. 3b–f). The effect size range of gRNA and gene level improved almost two-fold to 11.71 and 6.20 (from 6.47 and 3.19), respectively (Fig. 3b). Moreover, mean gene LFCs of core essential genes increased by three-fold to -3.69 (from − 1.15), which resulted in a total effect size range similar to single enAsCas12a and SpCas9 applications (enAsCas12a: − 3.56, SpCas9: -4.29, Fig. 3f–h). This vast improvement was likely the result of dual-gRNA gene targeting, which compensates for gRNA processing. Indeed, CHyMErA.v2-enAsCas12a gRNAs that targeted CE genes displayed indistinguishable mean LFC values as gRNAs derived from the single gRNA enAsCas12a screen (enAsCas12a: − 2.95, CHyMErA.v2(enAsCas12a): − 2.91) (Fig. 3i). However, despite the improvement of SpCas9 gRNAs within CHyMErA.v2, when compared to CHyMErA (Fig. 3j), Nothern Blotting of total gRNA transcripts revealed different RNA processing that potentially prevented the SpCas9 gRNAs within CHyMErA.v2 to perform as the gRNAs from the single SpCas9 library (SpCas9: − 3.29, CHyMErA.v2(SpCas9): − 1.39) (Fig. 3k). Moreover, the lower editing efficiency of the SpCas9 gRNA of CHyMErA.v2 (34.1%), when compared to SpCas9 (92.2%), was confirmed by independent TIDE analyses (Supplementary Fig. S10b), which supports the hypothesis of differences in RNA processing of hybrid gRNAs. In line with this, Northern Blotting again revealed a difference between RNA processing of gRNAs derived from CHyMErA.v2 or single SpCas9 and enAsCas12a gRNAs (Supplementary Fig. S10a). In agreement with our initial hypothesis, we observed different abundances of full-length (182 nts) and SpCas9-separated CHyMErA.v2 RNA transcript (86 nts) (Supplementary Fig. S10a), suggesting that the separation of SpCas9 gRNAs from hybrid or chimeric gRNAs, while efficient, potentially still is the rate-limiting step for SpCas9 gRNA activity in CHyMErA constructs. To complement this finding, we performed AU-ROC analysis of the screening data to quantify their individual performance in calling CE and TS genes. This revealed an overall comparable performance between enAsCas12a(dual-gRNA) and CHyMErA.v2 plasmids (AU-ROC; SpCas9: 0.84/0.96, enAsCas12a(dual-gRNA): 0.77/0.94 and CHyMErA.v2: 0.81/0.96) (Fig. 3l and Supplementary Fig. S6h). Together, these data demonstrated that enAsCas12a dual-gRNAs greatly improved the performance of CHyMErA.v2 and that SpCas9 gRNAs remained limited in their activity due to their need for RNA processing.

### Robust and efficient orthogonal CRISPR screens with multiSPAS

**Figure 4:**
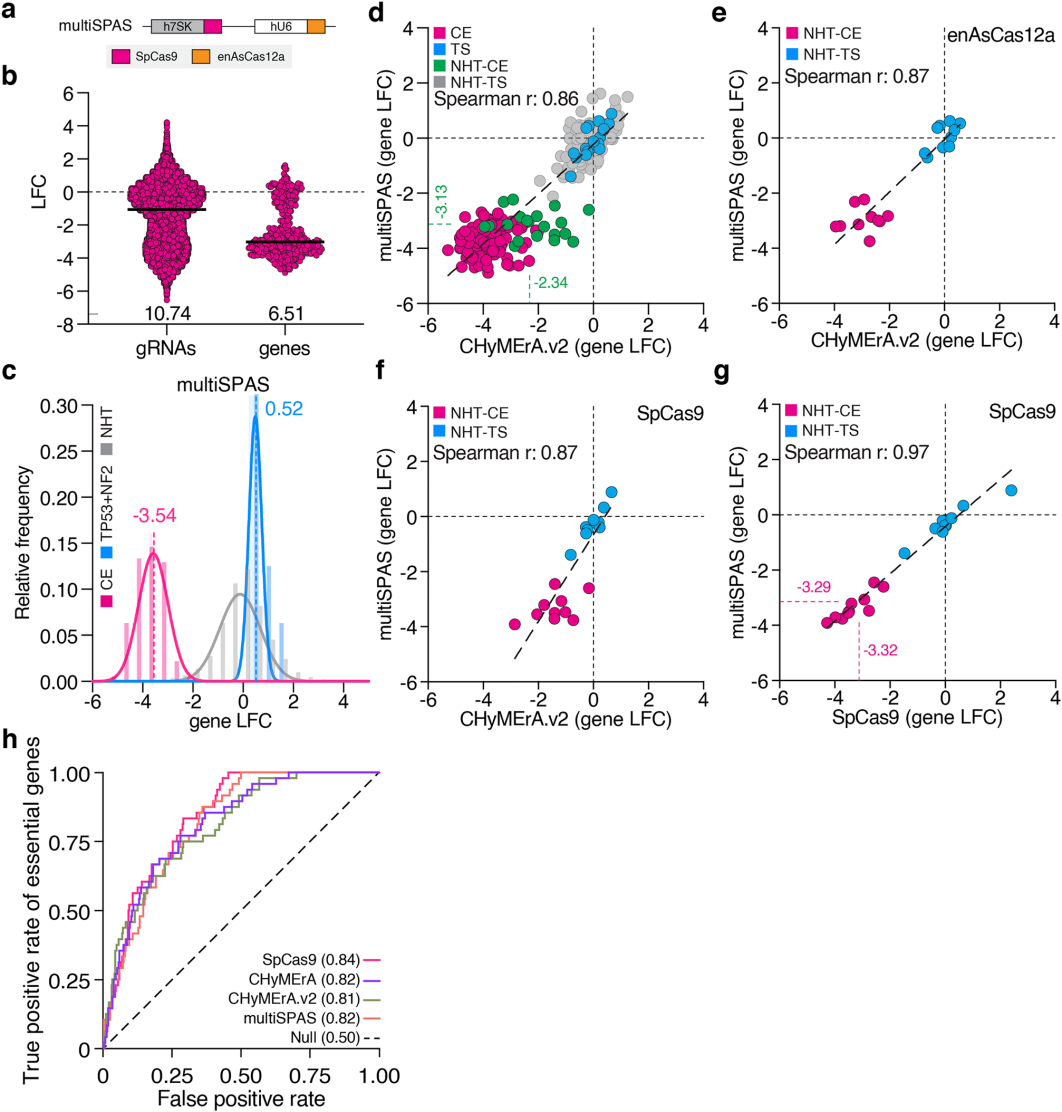
multiSPAS is a robust orthogonal screening approach. (**a**) Scheme of the multiSPAS gRNA expression vector. SpCas9 and enAsCas12a gRNAs are expressed from different promoters. (**b**) gRNA (left) and gene (right) level LFC distribution of multiSPAS screen. Numbers show the effect size range of the LFC values. Black lines represent the median value. (**c**) Density plot showing the relative frequency of gene level LFC. CE genes are indicated in pink, NHT in gray, and *TP53* and *NF2* in blue. The solid lines represent the nonlinear fit of the histogram and the bars represent the raw distribution. (**d**) Comparison of CHyMErA.v2 and multiSPAS gRNA level LFCs. (**e**) Gene level LFC comparison of enAsCas12a gRNAs and (**f**) SpCas9 gRNAs from CHyMErA.v2 and multiSPAS screens. (**g**) Gene level LFC comparison of SpCas9 gRNAs from SpCas9 and multiSPAS screens. Numbers are the median LFC value of CE-CE combinations. (**h**) Receiver operating characteristic (ROC) curves for guides targeting CE genes for SpCas9, CHyMErA, CHyMErA.v2, and multiSPAS screens. The area under the ROC curve (AU-ROC) values are given in parentheses. (**d**) CE-CE pairs targeting two CE genes are labeled with pink, TS-TS pairs targeting two TS genes with blue, NHT-CE, and NHT-TS pairs targeting a single gene with green and gray respectively. (**e**–**g**) NHT-CE gRNA pairs targeting a CE gene are highlighted in pink, and NHT-TS gRNA pairs targeting a TS gene are in blue.

The use of different Cas nucleases within the same cell enables orthogonal CRISPR approaches^9,10^. However, the robust performance of these approaches depends on both nucleases and their associated gRNAs performing optimally. Our data demonstrated that CHyMErA.v2 generates an improved effect size range when compared to its original version, but also that the activity of CHyMErA-intrinsic SpCas9 gRNAs is low due to RNA processing. We, therefore, engineered the orthogonal multiplex SpCas9-enAsCas12a approach (multiSPAS) in which SpCas9 and enAsCas12a gRNA-expression are decoupled by the use of two separate promoters each driving a single gRNA (Fig. 4a). We then used this plasmid for combinatorial library generation and screening, targeting the above-mentioned CE and TS genes (Supplementary Fig. S7c,d). Replicate correlation on gRNA and gene counts, as well as gRNA LFC levels, was high (gRNA *r* = 0.94, gene *r* = 0.95, gRNA LFC *r* = 0.92) (Supplementary Fig. S8a-d), with gRNA and gene effect size range comparable to CHyMErA.v2 (gRNA: 10.74, gene: 6.51) (Fig. 4b). The overall agreement between CHyMErA.v2 and multiSPAS was also high on the gene LFC levels of depleted CE and TS (only *NF2* and *TP53*) genes (CE—v2: − 3.69, multiSPAS: − 3.54; TS–v2: 0.22, multiSPAS: 0.52) (Fig. 4c). In more detail, NHT-CE gRNA combinations benefited strongly from being expressed separately (v2: − 2.34, multiSPAS: − 3.13) (Fig. 4d), and enAsCas12a gRNAs, as part of CHyMErA.v2 or multiSPAS, resulted in similar gene LFC values for the depletion of CE genes (Fig. 4e). This demonstrated that the single enAsCas12a gRNAs of multiSPAS performed comparably to the arrayed enAsCas12a gRNAs of CHyMErA.v2.

Next, we focused on analyzing the phenotypes of the SpCas9 gRNAs within multiSPAS, as these underperformed in previous orthogonal approaches. SpCas9 gRNAs of the multiSPAS plasmid showed a dramatic improvement in CE phenotypic separation, making them indistinguishable from the single SpCas9 library approach (mean LFC of CE genes; multiSPAS: -3.29, SpCas9: − 3.32) (Fig. 4f,g). This observation was further supported by an AU-ROC analysis comparing SpCas9, CHyMErA, CHyMErA.v2, and multiSPAS which revealed their overall similar performances in identifying CE and TS genes (AU-ROC; SpCas9: 0.84/0.96, CHyMErA: 0.82/0.92, CHyMErA.v2: 0.81/0.96 and multiSPAS: 0.82/0.94) (Fig. 4h and Supplementary Fig. S8e). Taking the improved effect size range of multiSPAS into account, this design was identified to be the most efficient among the tested orthogonal screening approaches. These data demonstrate that, by avoiding RNA hybrids, multiSPAS enables high performance of SpCas9 and enAsCas12a gRNAs for the identification of combinatorial genotype-to-phenotype associations. Moreover, generating libraries with large combinatorial diversities benefits from the use of independent oligo-pools that are combined in vitro, avoiding cost-ineffective pools with predefined gRNA combinations.

### enAsCas12a has a higher effect size range than opAsCas12a in multiSPAS screens

**Figure 5:**
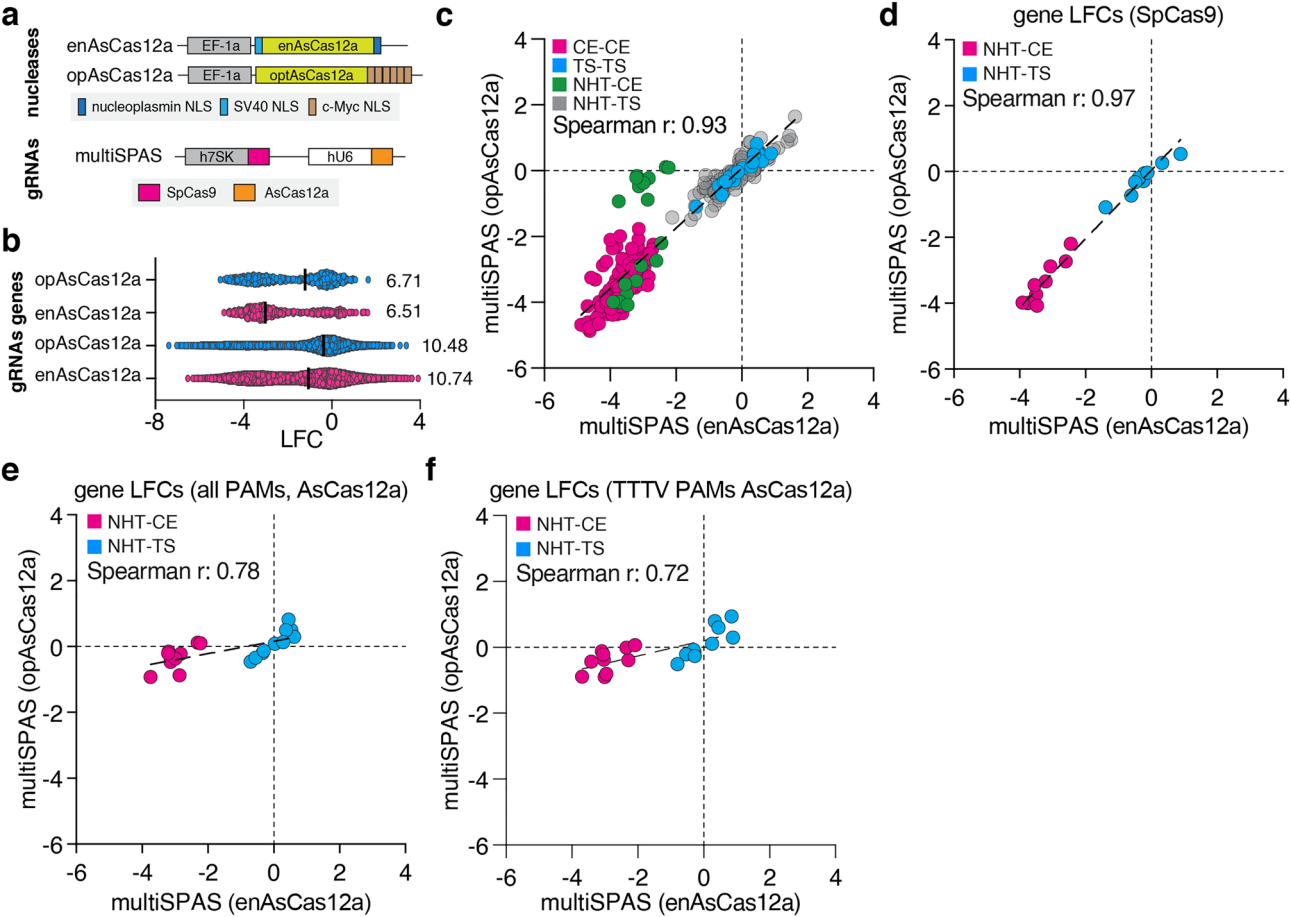
The effect size range of the multiSPAS is larger with enAsCas12a (**a**) Schematic representation of enAsCas12a and opAsCas12a nuclease expression constructs and scheme of the multiSPAS gRNA expression vector. (**b**) Comparison of gene level LFC (top) and gRNA level LFC (bottom) for multiSPAS(enAsCas12a) and multiSPAS(opAsCas12a). Lines represent the median LFC value. (**c**) Scatter plot comparing LFC of CE, TS, NHT-CE/TS gene pairs from multiSPAS(enAsCas12a) and multiSPAS(opAsCas12a) screens. CE-CE pairs targeting two CE genes are labeled with pink, TS-TS pairs targeting two TS genes with blue, and NHT-CE and NHT-TS pairs targeting a single gene with green and gray respectively. (**d**) Comparison of CE and TS gene level LFC for SpCas9 gRNAs and (**e**) AsCas12a (all gRNAs in the library) gRNAs from multiSPAS(enAsCas12a) and multiSPAS(opAsCas12a) screens. (**f**) Gene level LFC comparison of TTTV PAM-AsCas12a gRNAs from multiSPAS(enAsCas12a) and multiSPAS(opAsCas12a) screens. (**d–f**) NHT-CE gRNA pairs targeting a CE gene are highlighted in pink, and NHT-TS gRNA pairs targeting a TS gene are in blue. The dashed line is the linear regression line.

Optimized AsCas12a (opAsCas12a) is a rationally engineered version of AsCas12a, containing six c-terminal c-myc nuclear localization signals (NLSs) and two amino acid changes in the DNA binding domain, which was shown to have high-performance genome editing when targeting conserved protein domains^30^. Thus, we asked whether opAsCas12a would outperform enAsCas12a in orthogonal CHyMErA screens when gRNAs are designed based on an unbiased deep-learning approach. To investigate this, we used the above-described multiSPAS CRISPR library to target CE and TS genes in RPE1 cells stably expressing SpCas9 and opAsCas12a and compared the derived data to the enAsCas12a data set (Figs. 1b,c and 5a). The abundance of paired gRNAs was determined by deep-sequencing after 14 days of continuous cell culture, with library coverage maintained at 100-fold (Supplementary Fig. S9a). The high replicate correlation was observed for gRNA and gene read counts, as well as gRNA LFC levels (gRNA r = 0.96; gene r = 0.98; gRNA-LFC r = 0.90) (Supplementary Fig. S9b-d). When looking at gRNA and gene level effect size range first, no substantial difference between enAsCas12a and opAsCas12a could be observed (gRNAs/genes; enAsCas12a: 10.74/6.51, opAsCas12a: 10.48/6.71) (Fig. 5b). This finding was further supported by a high Spearman correlation of multiSPAS gRNA pairs (r = 0.93), as well as when comparing the activity of SpCas9 gRNAs under both conditions (SpCas9 gRNAs from enAsCas12a vs. opAsCas12a, r = 0.97) (Fig. 5c,d). However, when comparing the performance of Cas12a gRNAs among both enzymes, we observed an overall weaker performance of gRNAs when used by opAsCas12a, indicated by their relatively low correlation to enAsCas12a gRNAs (AsCas12a from enAsCas12a vs. opAsCas12a r = 0.78) (Fig. 5e). We observed comparable transcript and protein abundance of enAsCas12a and opAsCas12a (Supplementary Fig. S7a,b), which supports the conclusion that the improved performance of enAsCas12a is not related to a difference in protein abundance but rather a consequence of different editing efficiencies. Since opAsCas12a and enAsCas12a have different PAM requirements, with TTTV PAMs being efficiently used by both^30,31^, we repeated our analysis by only considering TTTV PAM-containing gRNAs. Still, opAsCas12a underperformed with respect to enAsCas12a, demonstrated by their relatively low correlation (AsCas12a TTTV-PAM gRNAs; r = 0.72) (Fig. 5f). Together, this demonstrates that when AsCas12a gRNAs are not designed to target conserved protein domains, enAsCas12a displays larger effect size range in drop-out CRISPR screens.

## Discussion

In the present study, we elucidated performance-critical parameters for combinatorial and orthogonal CRISPR screens. We compared combinatorial SpCas9, AsCas12a, and orthogonal CHyMErA screening approaches by targeting the same gene set under comparable experimental conditions and observed that single and paired gRNA effect size ranges are the largest with SpCas9. In line with our findings, the gRNA effect size range of AsCas12a was reported to be low^29^, and even though AsCas12a has been engineered and optimized to perform in CRISPR screens, also the recent literature indicates that AsCas12a is not as robustly performing as SpCas9^20,32^. However, the low effect size range associated with enAsCas12a and CHyMErA may be attributed to either difference in nuclease activity or the lack of respective highly-active spacer sequences. Despite SpCas9 and AsCas12a showing equivalent in vitro nuclease activity^33^, cellular applications with AsCas12a lag behind SpCas9 applications, which limits the performance of deep-learning gRNA design tools in finding highly-active AsCas12a gRNAs^14,34^. However, as more data on AsCas12a screens become available, AsCas12a gRNA design tools, and subsequently the quality of AsCas12a genetic screens, are expected to improve. We are aware that our conclusion of CHyMErA underperforming in combinatorial CRISPR screens contradicts previous observations^9^. These, however, may be explained by differences among the two used CHyMErA designs. In particular, the use of different Cas12a variants (As-Cas12a instead of Lb-Cas12a), varying numbers of SpCas9-coupled NLS sequences (this study uses 1 NLS while Gonatopoulos-Pournatzis et al. used 2 NLS), and the use of different Cas9 tracrRNAs (this study uses the wildtype sequence while Gonatopoulos-Pournatzis et al. used the engineered v2 sequence).gRNA combinations can be designed to target multiple genes at once or to target a single gene locus to increase the editing efficiency of the targeted gene. Targeting the same gene with multiple gRNAs increases the knockout efficiency, as has been shown for SpCas9 and AsCas12a applications^13,32,35^. Applying this concept, we engineered enAsCas12a(dual-gRNA) and CHyMErA.v2 library formats containing arrayed enAsCas12a gRNAs that target the same gene. Surprisingly, this strategy negatively impacted the enAsCas12a screens, while it enhanced the effect size range of CHyMErA applications. This difference may be explained two-fold: (i) the hybrid gRNA of CHyMErA folds so that the SpCas9 gRNA inhibits the first enAsCas12a gRNA, while the second enAsCas12a gRNA is free for binding and processing by enAsCas12a, and (ii) the different baseline performances of enAsCas12a and CHyMErA. Testing the first possibility will require additional experimental work. The second possibility is supported by the relatively high level of enAsCas12a performance (compared to SpCas9), while CHyMErA displayed only a marginal effect size range. This may be explained by the fact that the ratio of total to processed RNA in CHyMErA applications, irrespectively on the used Cas12a nuclease (Lb or As), is reported to be limited to 60%^9^. Although the performance improvements were seen with CHyMErA.v2, a closer look at individual gRNA activities revealed that SpCas9 gRNAs are mostly non-functional. This is well in line with the conclusion that the folding of hybrid gRNAs prevents the nuclease from accessing them, which also suggests that the CHyMErA.v2 phenotypes are likely driven by the second AsCas12a gRNA. Moreover, the improved effect of CHyMErA.v2 was limited to the targeting of core essential genes, which suggests the use of SpCas9 approaches when investigating positive gene fitness effects. Furthermore, even though library uniformity was previously predicted and shown to be critical for the experimental scale^13,36,37^, and since experimental parameters were kept consistent throughout our experiments, we rule out library distribution or coverage to confound the conclusion drawn here.

Our experiments and analysis identified AsCas12a dual-gRNAs to vastly underperform when compared to their sgRNAs counterparts. This observation was not restrictive to single, but also prominent in orthogonal applications. This is well in line with the literature in which the design and the sequence of the direct repeat, which separates two gRNAs in AsCas12a applications, influences the efficiency of gRNA processing^14,27^. Our presented library formats contain improved direct repeat sequences, thus, the underperformance of dual-gRNAs to sgRNAs is likely independent of the used direct repeats. Additionally, the use of different direct repeat variants avoids potential lentiviral recombination issues which can affect the performance of enAsCas12a(dual-gRNA) and CHyMErA.v2 in screens. However, since the design of our direct repeat does not contain a recently described separator sequence, which was demonstrated to improve RNA processing and downstream gRNA activity^27^, our combinatorial and orthogonal library formats containing AsCas12a dual-gRNAs can likely be improved further. opAsCas12a is an engineered AsCas12a version that contains six c-terminal c-myc nuclear localization signals (NLSs) and two amino acid changes in the DNA binding domain^30^. Recently, opAsCas12a was demonstrated to have comparable performance as SpCas9 in single gene knockout screens^30^. This is in contrast to our observation of SpCas9 working better than enhanced and optimized AsCas12a in orthogonal multiSPAS screens. While the different performances cannot be explained by restricting our analysis to TTTV PAMs, it may be explained by Gier et al. using an approach in which functional protein domains are targeted by multiple gRNAs, with their averaged effect serving as a surrogate for gene loss-of-function strength^30^. This strategy is well known to improve the knockout effect size range, even for SpCas9^38^. Since gRNAs in our study were designed by CRISPick^34^, which uses a deep-learning approach based on prior knowledge of functional gRNAs, the performance of multiSPAS with optimized AsCas12a may be improved by adapting gRNA sequences to target active domains. It should, however, be noted that this strategy is limited to genes for which functional domains are known, thus interfering with genome-scale applications.

In summary, our side-by-side comparison systematically investigates the performance of SpCas9, enhanced and optimized AsCas12a, as well as CHyMErA in combinatorial and orthogonal CRISPR screens, and identifies SpCas9 to provide the most robust effect size range across the tested conditions. While dual-gRNA targeting of single genes increases the performance of CHyMErA, SpCas9 gRNAs remain inactive due to the need for RNA processing. Despite this, we acknowledge that increasing the sample size of this study (e.g. number of tested genes and conditions) would likely increase the differences observed between the tested combinatorial plasmid designs. Combining these findings, we engineered multiSPAS, in which SpCas9 and AsCas12a gRNAs are physically separated which results in both of them being highly active in cells. This identified multiSPAS as a robust orthogonal CRISPR approach to studying genotype-to-phenotype associations.

## Methods

### Plasmid design

The information on each plasmid for combinatorial libraries is indicated in Supplementary Table S1. Plasmids used in this study to generate combinatorial libraries for SpCas9, enAsCas12a, CHyMErA, enAsCas12a (dual-gRNA), CHyMErA.v2, multiSPAS were deposited to Addgene (IDs: 189632, 189633, 189634, 189635, 189636, 189637, respectively).

### 3Cs oligonucleotide design

The protocol for 3Cs multiplex-DNA synthesis was adapted from Wegner et al*.*, Wegner et al*.* and Diehl et al.^13,21,39^. To enable specific annealing to either h7SK and hU6 (SpCas9, enAsCas12a, enAsCas12a(dual-gRNA), multiSPAS) or SpCas9 and AsCas12a (CHyMErA, CHyMErA.v2) gRNA expression cassettes, the 3Cs oligonucleotides were designed with two distinct homology regions which flank the dedicated 20 (for SpCas9) or 23 (enAsCas12a) nucleotide-long gRNA sequences. All homology sequences were extended to have an annealing temperature of 52–55 °C. When selecting active gRNA sequences, the top 4 highest ranked SpCas9 and enAsCas12a knockout gRNAs from CRISPick were selected^14,25,34,40^. For the dual-gRNA approaches (enAsCas12a(dual-gRNA) and CHyMErA.v2), first and fourth-ranked gRNAs or second and third-ranked gRNAs were chosen as pairs. gRNA sequences for single and pooled applications are listed in Supplementary Table S2 and S3.

### Preparation of dU-single-stranded DNA

50 ng of the 3Cs template plasmid were transformed into chemically competent K12 RecA knockout CJ236 *Escherichia coli (E. Coli)* strain and bacteria were grown on LB agar plates containing chloramphenicol (25 µg/ml) and ampicillin (100 µg/ml) overnight at 37 °C. Single bacteria colonies were picked and grown in 1 ml of 2xYT medium (Carl Roth GmbH, 6676.2) supplemented with ampicillin (100 µg/ml) and 1 × 10^8^ pfu of M13KO7 helper phage (New England Biolabs, N0315). After incubating at 200 rpm and 37 °C for 3 h, kanamycin (50 µg/ml) was added to select the M13KO7 helper phage-infected bacteria. After incubation of 6–8 h at 200 rpm and 37 °C, bacteria were transferred to 30 ml of 2xYT medium supplemented with chloramphenicol (25 µg/ml), ampicillin (100 µg/ml), and kanamycin (50 µg/ml). After 20 h of incubation at 200 rpm and 37 °C, the bacterial culture was centrifuged in a Beckman JA-12 fixed angle rotor for 10 min at 12,000*g* at 4 °C. The supernatant was transferred into a new tube with 6 ml PEG/NaCl buffer (20% polyethylene glycol 8000, 2.5 M NaCl) and incubated for 60 min at room temperature. The phage-containing mixture was centrifuged for 10 min at 12,000*g* at 4 °C. The supernatant was removed and the phage pellet was resuspended in 1 ml Dulbecco’s phosphate-buffered saline (PBS, Sigma-Aldrich, D8662) and centrifuged for 5 min at 16,000*g*. Phage-containing supernatant was transferred to a new tube and stored at 4 °C until single-stranded DNA (ssDNA) was purified. The circular ssDNA was isolated using the E.Z.N.A. M13 DNA Mini Kit (Omega Bio-Tek, D69001-01) according to the manufacturer’s protocol. The purity of ssDNA was controlled with agarose gel electrophoresis.

### Phosphorylation and annealing of oligonucleotides

0.6 µg of oligonucleotide per annealing site were phosphorylated separately. In a 1, 5 mL microcentrifuge tube the respective oligonucleotide, 2 µl of 10xTM Buffer (0.1 M MgCl_2_, 0.5 M Tris–HCl, pH 7.5), 2 µl of 10 mM ATP (New England Biolabs, 756), 1 µl of 100 mM DTT (Cell Signaling Technology Europe, 7016) and 2 µl of 10,000 U/ml T4 Polynucleotide Kinase (New England Biolabs, M0201) and water were mixed in a total volume of 20 µl. This mixture was incubated at 37 °C for 1 h. 20 µg of the ssDNA were mixed with 25 µl of 10 × TM buffer, 20 µl of each phosphorylated oligonucleotide, and water in a total volume of 250. This mixture was denatured, annealed, and cooled down at 90 °C for 5 min, 55 °C for 5 min, and room temperature for 10 min respectively.

### Synthesis of 3Cs-dsDNA

To the annealed ssDNA-oligonucleotide mix 10 µl of 10 mM ATP, 10 µl of 100 mM dNTP mix (Carl Roth GmbH, 0178.1/2), 15 µl of 100 mM DTT, 2000 ligation units of T4 DNA ligase (New England Biolabs, M0202) and 30 units of T7 DNA polymerase (New England Biolabs, M0274) were added. This reaction was incubated overnight at room temperature. 3Cs-reaction was purified by DNA Clean & Concentrator-25 kit (Zymo Research, D4034) according to the manufacturer's protocol. The cleaned-up 3Cs reaction product was analyzed on 0.8% agarose gel together with ds and ss-circular DNA.

### Electroporation and determination of transformation efficiency

All cleaned-up 3Cs synthesis product was transformed into 400 µl of electrocompetent *E. coli* (10-beta, New England Biolabs, C3020K) cells with Bio-Rad Gene Pulser (voltage 2.5 kV, capacitance 25 µF and resistance 200 Ω). The cells were immediately rescued with 20 ml prewarmed 2xYT medium. Cells were transferred into an Erlenmeyer flask and incubated at 37 °C and 200 rpm for 30 min. After 30 min, transformed bacteria were transferred into 200 ml of 2xYT medium with 100 μg/ml ampicillin and shaken overnight at 200 rpm and 37 °C. The number of transformed bacteria was determined to ensure library representation at least 100-fold higher than the library complexity. Transformed bacteria were serially diluted in sterile PBS and were plated in triplicates on 100 μg/ml ampicillin-containing LB agar plates and incubated overnight at 37 °C. The obtained colonies were counted to determine the number of transformants.

### Removing the residual template plasmid and quality control

Plasmid DNA was isolated from the overnight culture to obtain the pre-library using the GeneJet Plasmid Miniprep Kit (Thermo Fisher Scientific, K0503) according to the manufacturer's protocol. 3 µg of pre-library was digested with PacI and I-SceI (New England Biolabs, R0547S and R0694S) restriction enzymes to remove the residual template plasmid from the library which has recognition sequences in the wild-type gRNA placeholder at 37 °C overnight. Digested pre-library was purified using the DNA Clean & Concentrator-5 kit (Zymo Research, D4014), as described in the manufacturer’s protocol. The digested and purified pre-library was electroporated as described before for the electroporation of 3Cs reaction products. The next day the DNA was isolated using the QIAGEN Plasmid Maxi Kit (Qiagen, 12,163) according to the manufacturer's protocol. The purified library quality was assessed by next-generation sequencing.

### Plasmid library high-throughput sequencing

The plasmid libraries were prepared for Illumina high-throughput sequencing using PCR under the following conditions. 100 ng of the plasmid DNA was mixed with 25 µl of NEBNext High-Fidelity 2X PCR Master Mix (New England Biolabs, M0541) and 2.5 µl each primer (10 µm), then water was added to a final volume of 50 µl. PCR was performed using the following conditions: initial denaturation at 98 °C for 3 min, 20 cycles of denaturation at 98 °C for 30 s, annealing at 68 °C for 30 s and elongation at 72 °C for 30 s, and final extension at 72 °C for 5 min. The PCR products were purified from 1% agarose gel using the GeneJet Gel Extraction Kit (Thermo Fisher Scientific, K0692) according to the manufacturer’s protocol. Purified amplicons were then quantified using the Qubit fluorometer (Thermo Fisher Scientific). Gel-purified PCR products of plasmid libraries and screening samples were denatured and diluted according to Illumina guidelines and set to a final concentration of 16 pM in a total volume of 600 µl and 15% PhiX control and loaded onto a MiSeq sequencer (Illumina) according to the manufacturer’s protocol. Paired-end sequencing was performed, with 75 cycles per read and 8 cycles for the index read.

### Screen sample high-throughput sequencing

First, the amount of genomic DNA (gDNA) needed for adequate coverage was determined by calculating the “library complexity x experimental coverage × 6.6 pg”. The PCR amplification was performed in two steps. The calculated total amount of gDNA was used in the first PCR (PCR1) as 2 µg of gDNA for each reaction which was set up in 50 µl with 25 µl of NEBNext High-Fidelity 2X PCR Master Mix, 2.5 μl of each primer (10 μm) and water. The thermal cycler parameters were set as follows: the initial denaturation at 98 °C for 5 min, 20 cycles of denaturation at 98 °C for 50 s, annealing at 68 °C for 45 s, and elongation at 72 °C for 1 min, and final extension at 72 °C for 5 min. All PCR products were then pooled and mixed in the second PCR (PCR2). 25 µl of the PCR1 product was added into the PCR2 reaction together with 50 µl of NEBNext High-Fidelity 2X PCR Master Mix, 5 μl of each primer (10 μl) which contains Illumina adaptors and index sequences, and water up to 100 µl. The PCR2 was set as follows: initial denaturation at 98 °C for 3 min, 10 cycles of denaturation at 98 °C for 50 s, annealing at 68 °C for 45 s, and elongation at 72 °C for 45 s, and final extension at 72 °C for 5 min. PCR2 product was purified from 1% agarose gel and sequenced as described for the plasmid library. Primer sequences for PCR1 and PCR2 for all library constructs are listed in Supplementary Table S4.

### High-throughput sequencing data quality control, read count table generation, and analysis

Read counts of individual gRNA combinations were determined using cutadapt 2.8 and Bowtie 2.3.0^41,42^. Reads were trimmed with cutadapt in paired-end mode to the length of the respective library sequences using the constant regions upstream of the gRNA. Cutadapt was set up with an error tolerance of 0.1 and to discard untrimmed reads. The obtained sequences were then aligned to the respective gRNA library using Bowtie2 in local mode using a seed length of 11, a seed mismatch value of 1, and the interval function ‘S,1.0,0.75’. To assess the uniformity of each library, the read counts were plotted with a Lorenz curve as a cumulative distribution. According to Imkeller et al. the library skew was determined by dividing the 90th percentile by the 10th percentile of the read count distribution^36^. Obtained read counts for each individual gRNA combination were normalized to the total number of generated read counts and median read count of all NHT-NHT combinations within that sample. Read counts for each screen and library are given in Supplementary Table S5. LFC values for each gRNA combination were calculated by *log*_*2*_*(screen sample normalized read count/library normalized read count)*. LFC values for gene–gene combination were calculated by median aggregation of every gRNA-gRNA LFCs for the related genes.

### Cell culture conditions

Cell culture work was performed as described previously^21^. In brief, HEK293T cells (ATCC, CRL-3216) were maintained in Dulbecco’s Modified Eagle’s Medium (DMEM, Thermo Fisher Scientific, 41965-039) supplemented with 10% fetal bovine serum (FBS, Thermo Fisher Scientific, 10270) and 1% penicillin–streptomycin (Sigma-Aldrich, P4333) at 37 °C with 5% CO_2_. On the other hand, SpCas9 and enAsCas12a or opAsCas12a expressing RPE1 cells were cultured in DMEM: Nutrient Mixture F-12 (DMEM/F12, Thermo Fisher Scientific, 11320-074) supplemented with 10% FBS, 1% penicillin–streptomycin and 0.01 mg/ml hygromycin B (Capricorn Scientific, HYG-H)) at 37 °C with 5% CO_2_. Mycoplasma contamination testing was performed immediately after the arrival of the cells and regularly during the experiments by using VenorGeM Classic (Minerva Biolabs GmbH, 11-1025) according to the manufacturer’s protocol.

### Cell line generation and testing

Puromycin-sensitive hTERT-RPE1 cells were provided by Andrew Holland. To generate the RPE1 cell line constitutively expressing SpCas9 and enAsCas12a or opAsCas12a; first, puromycin-sensitive RPE1 cells were transduced with lentiviral particles of the transfer plasmid lentiCRISPRv2-neo (Addgene: 98292). Then, a single clone with high SpCas9 activity was selected and transduced with lentiviral particles generated with the pRDA_174 (Addgene: 136476) enAsCas12a or pRG232 (Addgene: 149723) opAsCas12a expressing construct. Clones of SpCas9- and enAsCas12a-expressing RPE1s were derived and tested for enAsCas12a activity by Nutlin-3 (Selleckchem, S8059) treatment, coupled to proliferation assay upon CRISPR-mediated *TP53* knockout.

### Extracting gDNA

Cells were lysed in 12 mL of TEX buffer (10 mM Tris–HCl, pH 7.5; 1 mM EDTA, pH 7.9; 0.5% SDS) containing ribonuclease A (RNase, 20 mg/mL, Carl Roth GmbH, 7156.2) and proteinase K (20 mg/mL, Carl Roth GmbH, 7528.6) and incubated overnight at 37 °C under constant agitation. The next day after complete lysis, 4 mL of 5 M NaCl was added, and the mixture was shaken vigorously and incubated at 4 °C for 1 h. The tube was centrifuged at 15,000*g* for 1 h at 4 °C. The supernatant containing the DNA was transferred into a new tube. 24 mL of ice-cold absolute ethanol was added, and mixed and the solution was incubated overnight at − 20 °C. The next day, the tube was centrifuged at 15,000*g* for 1 h at 4 °C. The supernatant was removed and the DNA pellet was washed with 10 mL of ice-cold 70% ethanol and mixed very well. The mixture was centrifuged at 15000*g* for 1 h at 4 °C and the supernatant was removed. Air-dried DNA pellets were resuspended in 1 mL of sterile water.

### Generation and quantification of lentiviral particles

To generate the lentiviral particles 4 × 10^6^ HEK293T cells were seeded in a 10 cm dish 1 day before the transfection. On the day of transfection 2 mL Opti-MEM I (Thermo Fisher Scientific, 31985062), 210 µl of GeneJuice transfection reagent (Sigma Aldrich, 70967-4), 33 µg of the transfer vector, 27 µg of psPAX2 (Addgene: 12260) and 10 µg of pMD2.G (Addgene: 12259) were mixed. This mixture was generously vortexed and incubated for 30 min at room temperature. After incubation, the mixture was added dropwise to the cells. The supernatant was collected 48 h after the transfection and stored at -80 °C. To determine the titer of the lentiviral particles, 50,000 RPE1 cells were seeded in two 6-well plates in 2 ml of DMEM/F12 together with 8 µg/ml polybrene (Sigma-Aldrich, H9268). Cells were transduced with a ten-fold dilution series of viral supernatant (10^2^ to 10^11^). Two days after the transduction, cells were selected with puromycin (1 µg/ml, InvivoGen ant-pr-5). Established colonies were counted 10–14 days after the transduction and the colony number in the highest dilution was volume normalized to determine the lentiviral titer.

### CRISPR screening

All combinatorial CRISPR libraries were screened in RPE1-SpCas9-enAsCas12a or SpCas9-opAsCas12a cell lines with 100-fold coverage, each with two technical replicates. The total cell number for each screen was calculated by multiplying library complexity and the coverage of 100. A total of 6 million cells (for SpCas9, en/opAsCas12a, CHyMErA, multiSPAS libraries) or 3 million cells (for CHyMErA.v2 library) or 1.5 million cells (for enAsCas12a(dual-gRNA) library) were transduced with a respective library with MOI of 0.5. Cells were selected with puromycin (1 µg/ml) 2 days after the transduction. Two days later, the culture medium was replaced with a fresh puromycin-containing medium, and cells were cultured in a medium with puromycin until the end of the screen. Cells were subcultured and seeded at a cell number maintaining library diversity (library complexity x screening coverage) when they reached 80% confluency. Cells were harvested after 10 cell doublings, on average 14 days, after the transduction for subsequent gDNA extraction.

### DNA oligonucleotides and oligonucleotide pools

Gene blocks, oligonucleotide pools, and oligonucleotides used for 3Cs reactions, cloning, PCR, and sequencing libraries were obtained from Integrated DNA Technologies (IDT) or Merck KGaA.

### RPE1 SpCas9 and enAsCas12a activity

SpCas9 and enAsCas12a activity of RPE1 cells was tested by Nutlin-3 treatment upon TP53 knockout, coupled with viability assay. First, we generated TP53 targeting gRNA constructs for both SpCas9 and enAsCas12a. RPE1 cells were seeded in a 48-well plate (1000 cells per well) and transduced with SpCas9-TP53 or enAsCas12a-TP53 or SpCas9-AAVS1 lentiviral particles. The next day, transduced cells were selected with puromycin (1 µg/ml). Three days after the transduction, cells were treated either with DMSO or Nutlin-3 (Selleckchem, S8059) (1 µM). Cell viability was recorded with alamarBlue (BioRad, BUF012A) according to the manufacturer's protocol for 14 days. gRNA sequences for the Cas activity experiment are listed in Supplementary Table S2.

### enAsCas12a processing activity

SpCas9-TP53 and enAsCas12a-HPRT1 targeting gRNAs were cloned into a single gRNA expressing plasmid (lentiCRISPRv2 for SpCas9 and pRDA_052 for enAsCas12a) which does not require gRNA processing and into CHyMErA plasmid which requires gRNA processing by enAsCas12a. In a 48-well plate, 1000 RPE1 cells were seeded and transduced with lentiviral particles of dual-gRNAs, sgRNAs, or NHT gRNAs. The next day, cells were selected with puromycin (1 µg/ml). Three days after the transduction, cells were treated either with DMSO or Nutlin-3 and 6-Thioguanine (Sigma Aldrich, A4882) (1 µM and 2.5 µM respectively). Cell viability was recorded with alamarBlue (BioRad, BUF012A) according to the manufacturer’s protocol for 20 days. gRNA sequences for processing activity experiment are listed in Supplementary Table S2.

### TIDE assay

To quantify the editing efficiency of the different gRNA expression constructs, the TIDE (Tracking of Indels by decomposition) assay was performed. The gRNAs targeting the AAVS1 locus were designed using the CRISPick^14,25,34,40^. The top-ranked gRNA for SpCas9 and enAsCas12a were selected and cloned into the h7SK and hU6 gRNA expression cassettes of every plasmid used in this study (SpCas9, enAsCas12a, CHyMErA, enAsCas12a (dual-gRNA), CHymErA.v2, multiSPAS) with 3Cs multiplex-DNA synthesis protocol which was previously explained in detail. In a 6-well plate, 50,000 RPE1 cells were seeded and transduced with lentiviral particles of AAVS1 gRNA-expressing constructs as well as the NHT control construct. Cells were selected with puromycin (1 µg/ml) 2 days after the transduction and kept in a medium with puromycin until they were harvested on day 8 after the transduction. The gDNA was isolated by PureLink genomic DNA isolation kit (Invitrogen, K182001) according to the manufacturer’s protocol. 200 ng of gDNA were mixed with 2.5 µl of forward and reverse primer mix (10 µM) and 25 µl of NEB Next Ultra II Q5 Master mix (New England Biolabs, M0554X) to amplify the targeted AAVS1 locus. PCR was performed with the following parameters: initial denaturation at 98 °C for 3 min; 30 cycles of denaturation at 98 °C for 30 s, annealing at 66 °C for 30 s, and elongation at 72 °C for 30 s, and final extension at 72 °C 5 min. The PCR product was purified by DNA Clean & Concentrator Kit 25 (Zymo Research, D4034) and 120 ng of the purified product was Sanger sequenced (Microsynth) with the forward PCR primer.Ultimately, the sequencing results were analyzed with TIDE^26^ to calculate total editing efficiencies for each construct. Artificial SpCas9 gRNA sequence that has the same target locus as the used enAsCas12a AAVS1 gRNA because TIDE allows only the NGG-PAM Cas9 gRNA input.

### SDS-PAGE and immunoblotting

Protein extracts were prepared by washing the cells with PBS and adding ice-cold lysis buffer, containing 50 mM Tris–HCl pH = 8.1, 150 mM NaCl, 1% NP40, 0.5% DOC, 0.1% SDS, 1 µg/ml benzonase (Sigma Aldrich, E1014), 50 U/ml RNase If (NEB, M0243S), 2 U/ml DNase I (NEB, M0303S) and protease inhibitors cocktail and incubated on ice for 15 min^43^. Extracts were maintained on ice for 10 min and centrifuged at 14,000 rpm at 4 °C for 10 min. The supernatants were collected and protein was quantified by the Pierce BCA Protein Assay Kit (Thermo Scientific, 23227). Samples were then denatured at 90ºC for 5 min. 35 µg of total protein and protein marker (AppliChem, A8889) were separated by 7.5% SDS-PAGE and transferred to Nitrocellulose membranes (Roti-NC 0.45 µm, Carl Roth GmbH, 9200.1). Membranes were blocked with 5% nonfat milk TBS-T (13 mM Tris, 60 mM NaCl, 0.5% Tween, pH 7.6) for 30 min at room temperature and incubated with the following primary antibodies overnight at 4 °C: 1:1000 mouse monoclonal anti-Cas9 (Santa Cruz Biotechnology, sc-517386) or 1:1000 mouse monoclonal anti-HA (clone 12CA5, Merck KGaA, 11583816001) or 1:1000 rabbit monoclonal anti-AsCas12a (Cell Signaling Technology, 19984). 1:5000 goat anti-mouse IgG HRP secondary antibody (Thermo Scientific, 31430) or 1:10000 IRDye 800CW goat anti-rabbit IgG secondary antibody (LI-COR Biosciences, 926-32211) were incubated for 1 h at room temperature. Bands were detected using Pierce ECL Western Blotting Substrate (Thermo Scientific, 32106) for the HRP antibody. Images were acquired using ChemiDoc MP Imaging System (Biorad, 1708280) or Odyssey DLx Imaging System (LI-COR Biosciences, 9142). Ponceau staining was performed to normalize protein expression.

### Northern blotting

RPE1 cells were transduced with AAVS1 or NHT gRNA expressing constructs (SpCas9 NHT, enAsCas12a NHT, SpCas9 AAVS1, enAsCas12a AAVS1, CHyMErA AAVS1, CHyMErA.v2 AAVS1, enAsCas12a(dual-gRNA) AAVS1, multiSPAS AAVS1). Transduced cells were selected with puromycin (1 µg/ml) for 4 days following the transduction and harvested on day 7 for subsequent RNA extraction according to the manufacturer’s protocol (Qiagen, 74134). For northern blotting analysis, 5 μg of each RNA were mixed with 2 × GLII loading buffer (NEB, B0363A), boiled at 95 °C for 5 min, and separated on an 8% polyacrylamide gel containing 7 M urea at 300 V for 140 min using a gel transfer system (Doppel-Gelsystem Twin L, PerfectBlue). RNA was transferred onto Hybond-XL membranes (BLOTTING-NYLON 66 MEMBRANES, TYPE B, POS., Sigma-Aldrich, 15356-1EA) using an Electroblotter with an applied voltage of 50 V for 1 h at 4 °C (Tank-Elektroblotter Web M, PerfectBlue), crosslinked with UV-light at 0.12 Joules (UV-lamp T8C; 254 nm, 8W), hybridized overnight in 15 ml Roti-Hybri-Quick buffer at 42 °C with 5 µl γ 32P-ATP end-labeled oligodeoxyribonucleotides and visualized on a Phosphorimager (Typhoon FLA 7000, GE Healthcare). gRNA and probe sequences for northern blot experiments are listed in Supplementary Table S2.

### qPCR

Cells were washed, trypsinized, and harvested at 80% confluency from a 6-well plate. RNA was extracted from the cell pellet using the RNeasy Plus Mini Kit (Qiagen, 74136) according to the manufacturer’s protocol. 10 µl of the RNA was subsequently reverse transcribed into cDNA using the High-Capacity cDNA Reverse Transcription Kit (Applied Biosystems, 4368814) following the manufacturer’s instructions. Finally, quantitative Polymerase Chain Reactions were performed in technical quadruplicates as follows: 1 µl of cDNA was added to 5 µl 2X PrimaQUANT qPCR-CYBR-Mastermix (Steinbrenner, SL-9902), 1 µl 10 µM forward and reverse primer mix and 3 µl ultrapure water. A specific pair of primers was designed for SpCas9, AsCas12a, and the GAPDH gene (Supplementary Table S2). Reactions were run on a Roche LightCycler-480-system and data was analyzed using the delta-delta-CT-method.

### ROC analysis

To compare the performance of different CRISPR screening approaches we performed area under the receiver operating characteristic curve (AU-ROC) analysis. By this, we evaluated their efficiencies in resembling fitness effects of gRNAs targeting either CE or TS genes. We used the median Chronos dependency score of RPE1 cell lines according to Cancer Dependency Map (DepMap) project to define the true positive and false positive rates. We establish a gold standard in which the positive class of CE genes (n = 6, namely *CCT4, EIF3B, XPO1, IARS, EFTUD2, NARS*) is represented with strong negative dependency scores that are conserved among RPE1 cell lines. Similarly, we defined the positive class of TS genes (n = 5, namely *ARNT, AHR, KIRREL, TP53, NF2*) with strong positive scores. The remaining genes of the library (n = 9) belong to the negative class of non-essential genes. The AU-ROC plots were generated using the pROC R package, which calculates AUC values based on the LFC of all sgRNAs targeting each of the 20 genes in the library.

## Supplementary Information

Supplementary Figure S1.

Supplementary Figure S2.

Supplementary Figure S3.

Supplementary Figure S4.

Supplementary Figure S5.

Supplementary Figure S6.

Supplementary Figure S7.

Supplementary Figure S8.

Supplementary Figure S9.

Supplementary Figure S10.

Supplementary Figure S11.

Supplementary Legends.

Supplementary Table S1.

Supplementary Table S2.

Supplementary Table S3.

Supplementary Table S4.

Supplementary Table S5.

## Data Availability

Illumina sequencing data are provided as raw read count tables in Supplementary Table S5. Additionally, the datasets generated during the current study are available in the GEO repository, via https://www.ncbi.nlm.nih.gov/geo/query/acc.cgi?acc=GSE215175 with private token “opqremuypputbmf”. New lentiviral 3Cs plasmids associated with this study will be made available from Addgene (IDs 189632 to 189637).

## References

[1] Dempster JM (2019). Agreement between two large pan-cancer CRISPR-Cas9 gene dependency data sets. Nat. Commun..

[2] Behan FM (2019). Prioritization of cancer therapeutic targets using CRISPR-Cas9 screens. Nature.

[3] Hustedt N (2019). A consensus set of genetic vulnerabilities to ATR inhibition. Open Biol..

[4] Meyers RM (2017). Computational correction of copy number effect improves specificity of CRISPR–Cas9 essentiality screens in cancer cells. Nat. Genet..

[5] Thompson NA (2021). Combinatorial CRISPR screen identifies fitness effects of gene paralogues. Nat. Commun..

[6] Parrish PCR (2021). Discovery of synthetic lethal and tumor suppressor paralog pairs in the human genome. Cell Rep..

[7] Ito T (2021). Paralog knockout profiling identifies DUSP4 and DUSP6 as a digenic dependence in MAPK pathway-driven cancers. Nat. Genet..

[8] Dede M, McLaughlin M, Kim E, Hart T (2020). Multiplex enCas12a screens detect functional buffering among paralogs otherwise masked in monogenic Cas9 knockout screens. Genome Biol..

[9] Gonatopoulos-Pournatzis T (2020). Genetic interaction mapping and exon-resolution functional genomics with a hybrid Cas9-Cas12a platform. Nat. Biotechnol..

[10] Najm FJ (2018). Orthologous CRISPR-Cas9 enzymes for combinatorial genetic screens. Nat. Biotechnol..

[11] Gilbert LA (2014). Genome-scale CRISPR-mediated control of gene repression and activation. Cell.

[12] Boettcher M (2018). Dual gene activation and knockout screen reveals directional dependencies in genetic networks. Nat. Biotechnol..

[13] Diehl V (2021). Minimized combinatorial CRISPR screens identify genetic interactions in autophagy. Nucleic Acids Res..

[14] DeWeirdt PC (2020). Optimization of AsCas12a for combinatorial genetic screens in human cells. Nat. Biotechnol..

[15] Han K (2017). Synergistic drug combinations for cancer identified in a CRISPR screen for pairwise genetic interactions. Nat. Biotechnol..

[16] Shen JP (2017). Combinatorial CRISPR–Cas9 screens for de novo mapping of genetic interactions. Nat. Methods.

[17] Wong ASL (2016). Multiplexed barcoded CRISPR-Cas9 screening enabled by CombiGEM. Proc. Natl. Acad. Sci. U. S. A..

[18] Chow RD (2019). In vivo profiling of metastatic double knockouts through CRISPR-Cpf1 screens. Nat. Methods.

[19] Aregger M, Xing K, Gonatopoulos-Pournatzis T (2021). Application of CHyMErA Cas9-Cas12a combinatorial genome-editing platform for genetic interaction mapping and gene fragment deletion screening. Nat. Protoc..

[20] Li R (2022). Comparative optimization of combinatorial CRISPR screens. Nat. Commun..

[21] Wegner M (2019). Circular synthesized CRISPR/Cas gRNAs for functional interrogations in the coding and noncoding genome. Elife.

[22] Brown KR, Mair B, Soste M, Moffat J (2019). CRISPR screens are feasible in *TP 53* wild-type cells. Mol. Syst. Biol..

[23] Vassilev LT (2004). In vivo activation of the p53 pathway by small-molecule antagonists of MDM2. Science.

[24] Kim E, Hart T (2021). Improved analysis of CRISPR fitness screens and reduced off-target effects with the BAGEL2 gene essentiality classifier. Genome Med..

[25] Sanson KR (2018). Optimized libraries for CRISPR-Cas9 genetic screens with multiple modalities. Nat. Commun..

[26] Brinkman EK, Chen T, Amendola M, van Steensel B (2014). Easy quantitative assessment of genome editing by sequence trace decomposition. Nucleic Acids Res..

[27] Magnusson JP, Rios AR, Wu L, Qi LS (2021). Enhanced Cas12a multi-gene regulation using a CRISPR array separator. Elife.

[28] McCarty NS, Graham AE, Studená L, Ledesma-Amaro R (2020). Multiplexed CRISPR technologies for gene editing and transcriptional regulation. Nat. Commun..

[29] Zetsche B (2017). Multiplex gene editing by CRISPR-Cpf1 using a single crRNA array. Nat. Biotechnol..

[30] Gier RA (2020). High-performance CRISPR-Cas12a genome editing for combinatorial genetic screening. Nat. Commun..

[31] Kleinstiver BP (2019). Engineered CRISPR-Cas12a variants with increased activities and improved targeting ranges for gene, epigenetic and base editing. Nat. Biotechnol..

[32] Liu J (2019). Pooled library screening with multiplexed Cpf1 library. Nat. Commun..

[33] Kocak DD (2019). Increasing the specificity of CRISPR systems with engineered RNA secondary structures. Nat. Biotechnol..

[34] Kim HK (2018). Deep learning improves prediction of CRISPR-Cpf1 guide RNA activity. Nat. Biotechnol..

[35] Campa CC, Weisbach NR, Santinha AJ, Incarnato D, Platt RJ (2019). Multiplexed genome engineering by Cas12a and CRISPR arrays encoded on single transcripts. Nat. Methods.

[36] Imkeller K, Ambrosi G, Boutros M, Huber W (2020). gscreend: Modelling asymmetric count ratios in CRISPR screens to decrease experiment size and improve phenotype detection. Genome Biol..

[37] Heo S-J (2022). Optimized CRISPR guide RNA library cloning reduces skew and enables more compact genetic screens. BioRxiv.

[38] Shi J (2015). Discovery of cancer drug targets by CRISPR-Cas9 screening of protein domains. Nat. Biotechnol..

[39] Wegner M, Husnjak K, Kaulich M (2020). Unbiased and tailored CRISPR/Cas gRNA libraries by synthesizing covalently-closed-circular (3Cs) DNA. Bio Protoc..

[40] Doench JG (2016). Optimized sgRNA design to maximize activity and minimize off-target effects of CRISPR-Cas9. Nat. Biotechnol..

[41] Langmead B, Salzberg SL (2012). Fast gapped-read alignment with Bowtie 2. Nat. Methods.

[42] Martin M (2011). Cutadapt removes adapter sequences from high-throughput sequencing reads. EMBnet J..

[43] Kaulich M (2015). Efficient CRISPR-rAAV engineering of endogenous genes to study protein function by allele-specific RNAi. Nucleic Acids Res..

