# Supplementary figures and images for: Optimized metrics for orthogonal combinatorial CRISPR screens
Source: Sci Rep. 2023 May 6;13:7405. doi: 10.1038/s41598-023-34597-8 (PMC10164157; doi:10.1038/s41598-023-34597-8)

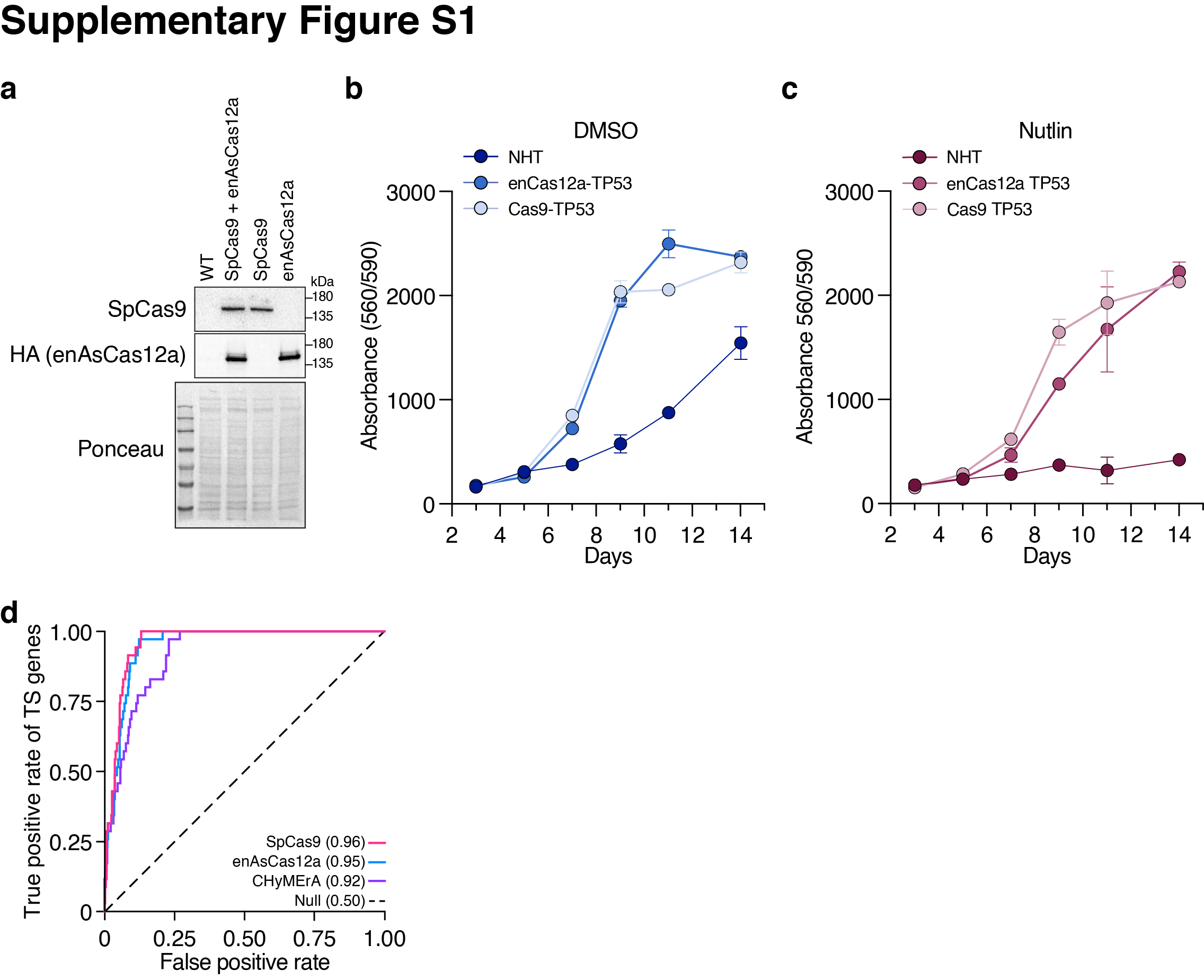

Supplement: Supplementary file 1 — Supplementary Figure S1. [file 41598_2023_34597_MOESM1_ESM.png]

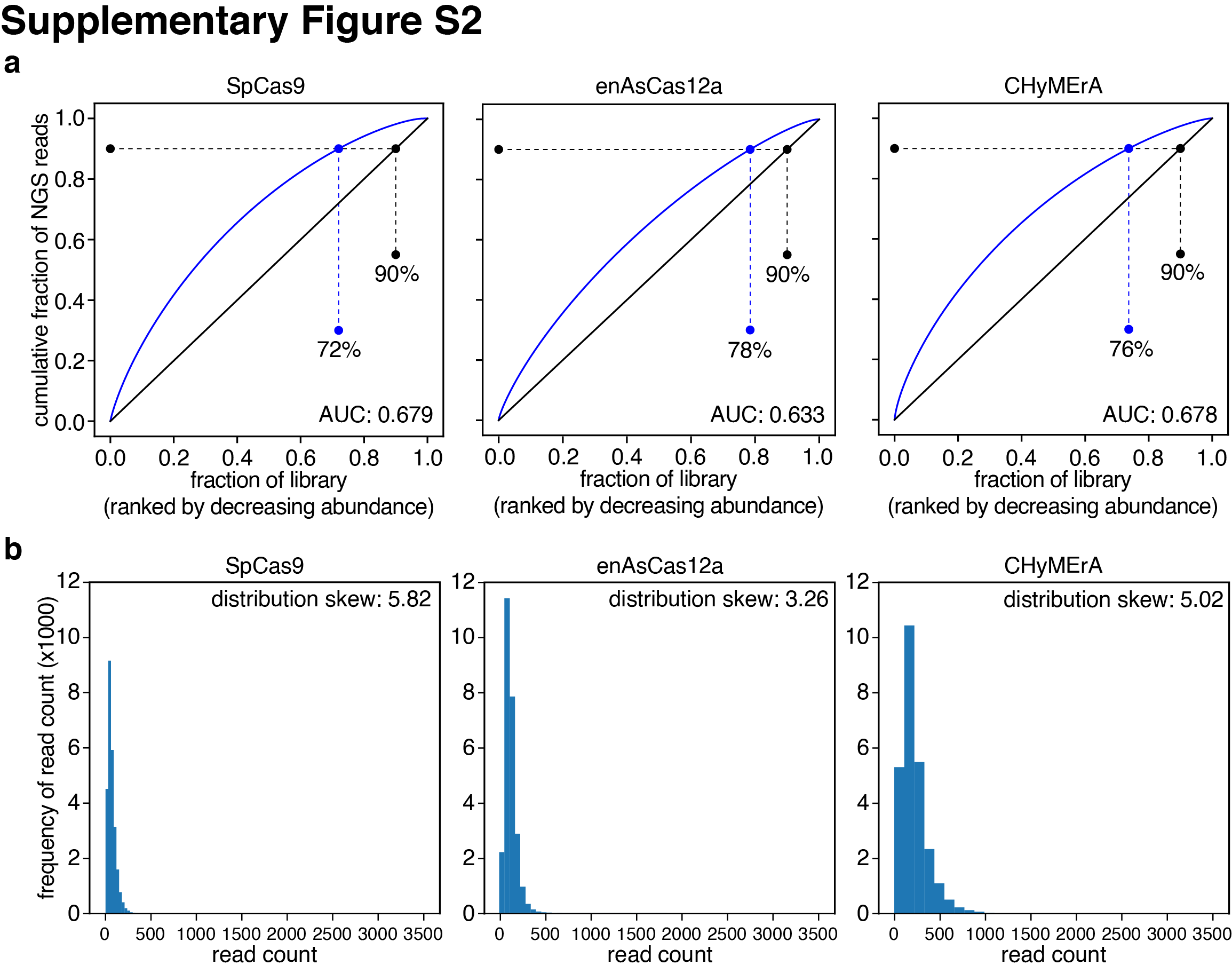

Supplement: Supplementary file 2 — Supplementary Figure S2. [file 41598_2023_34597_MOESM2_ESM.png]

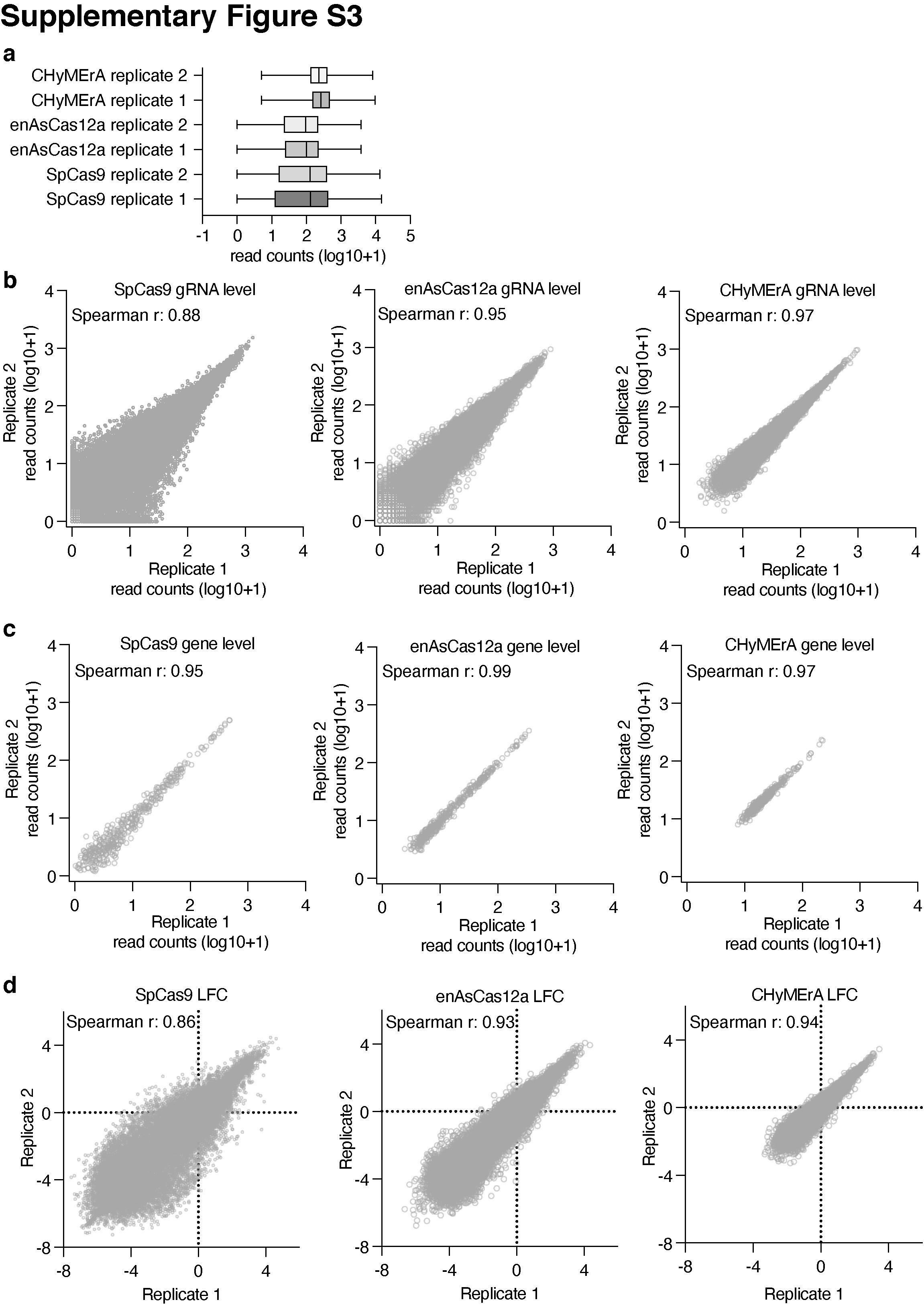

Supplement: Supplementary file 3 — Supplementary Figure S3. [file 41598_2023_34597_MOESM3_ESM.png]

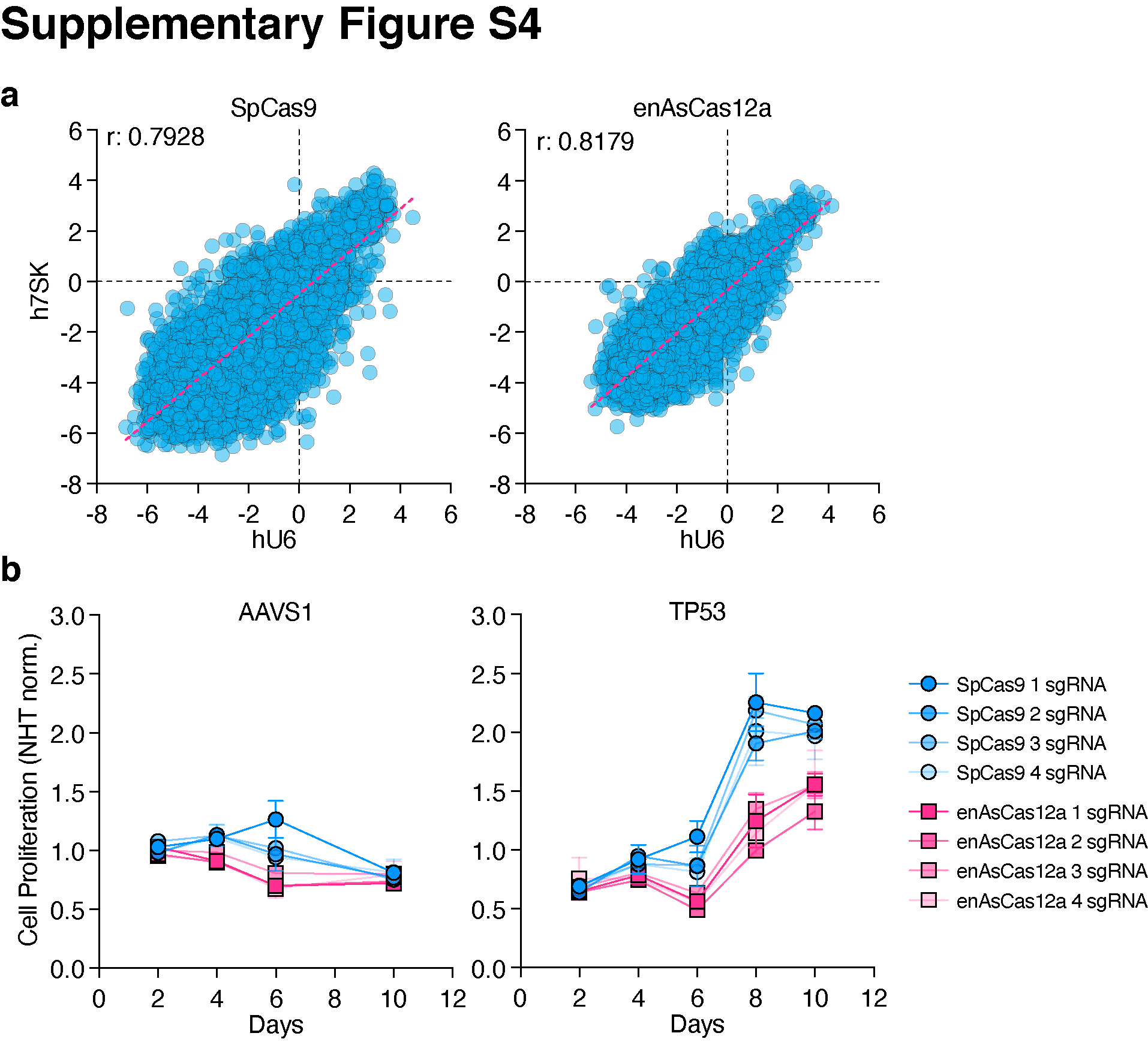

Supplement: Supplementary file 4 — Supplementary Figure S4. [file 41598_2023_34597_MOESM4_ESM.png]

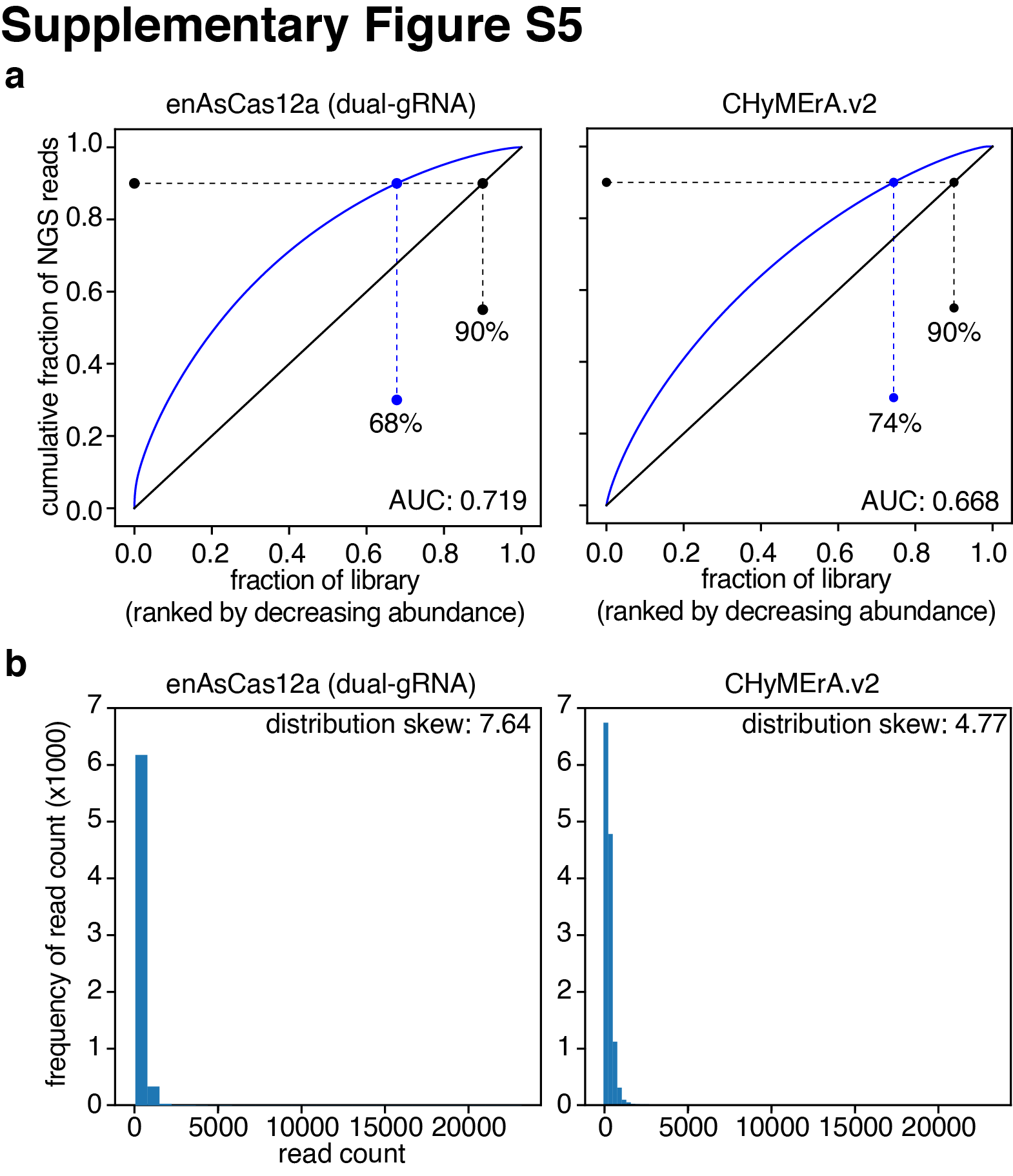

Supplement: Supplementary file 5 — Supplementary Figure S5. [file 41598_2023_34597_MOESM5_ESM.png]

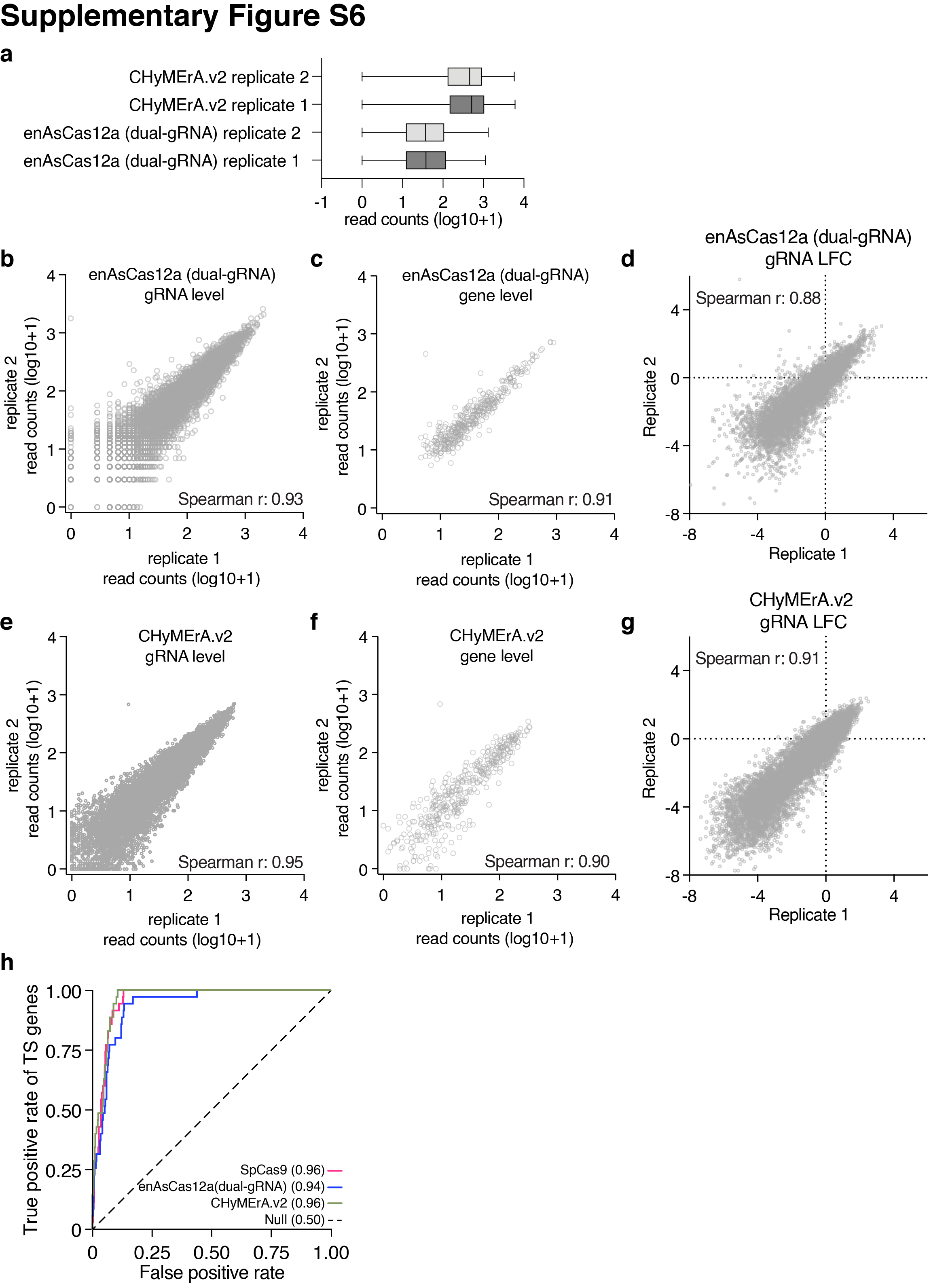

Supplement: Supplementary file 6 — Supplementary Figure S6. [file 41598_2023_34597_MOESM6_ESM.png]

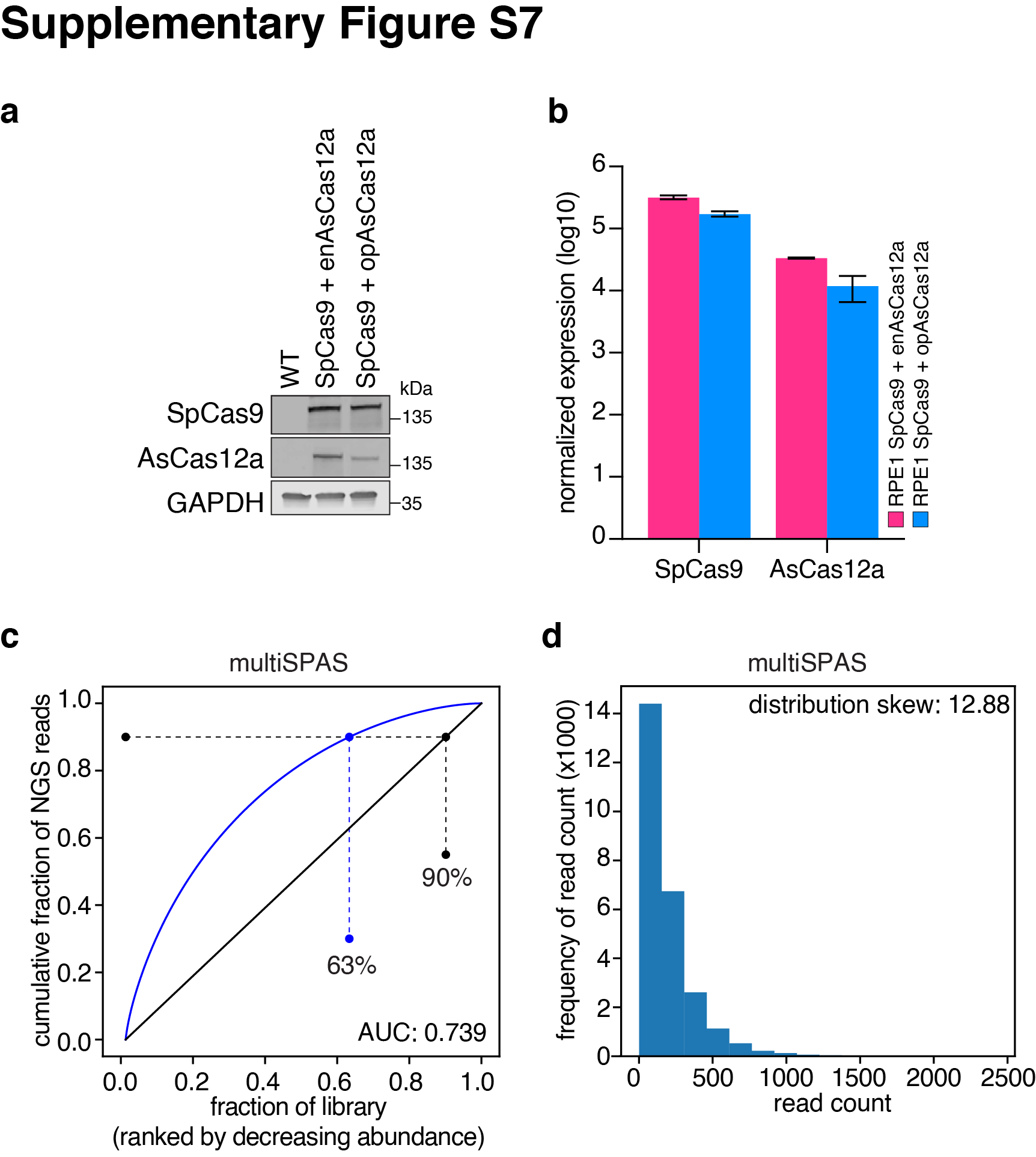

Supplement: Supplementary file 7 — Supplementary Figure S7. [file 41598_2023_34597_MOESM7_ESM.png]

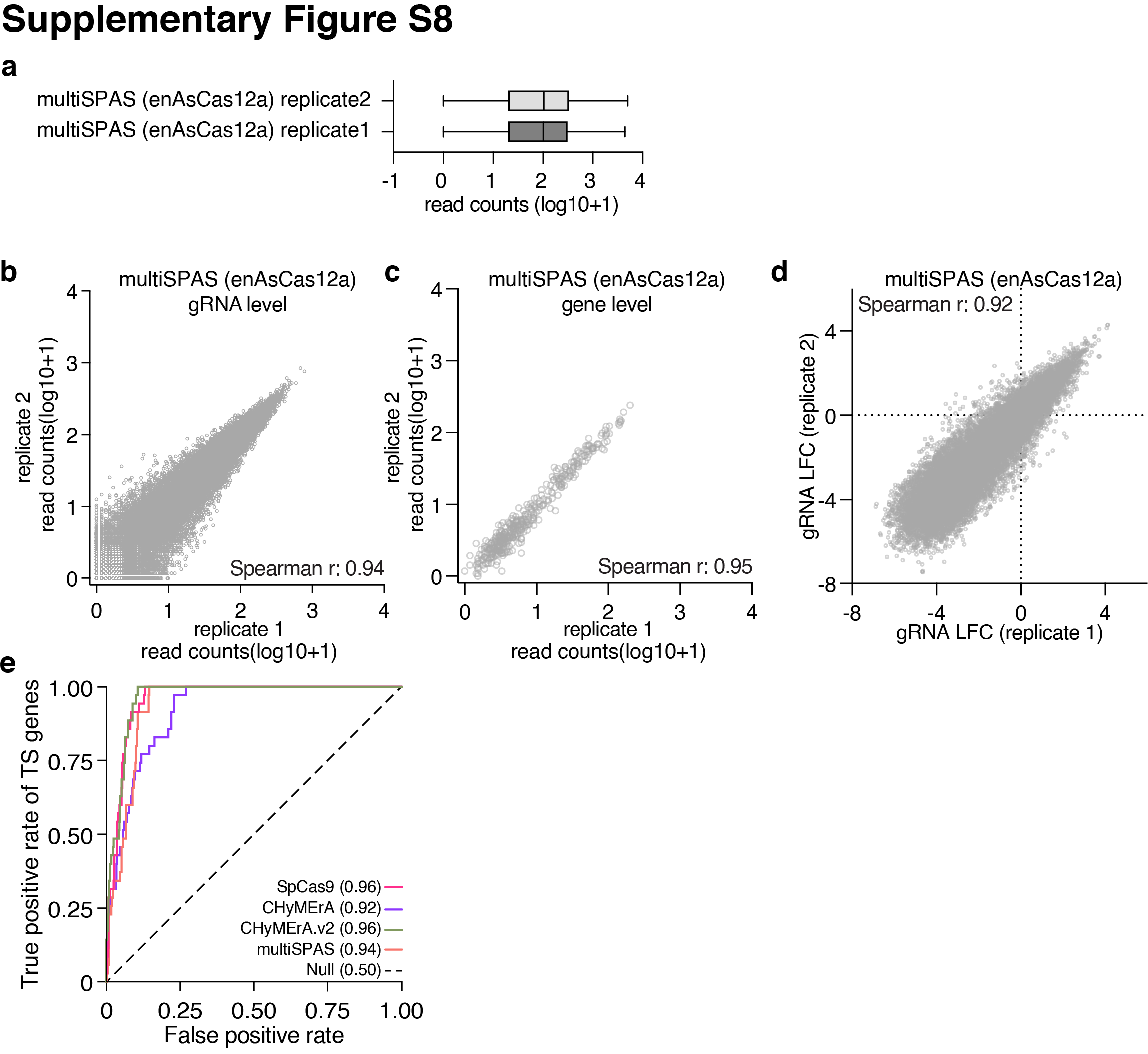

Supplement: Supplementary file 8 — Supplementary Figure S8. [file 41598_2023_34597_MOESM8_ESM.png]

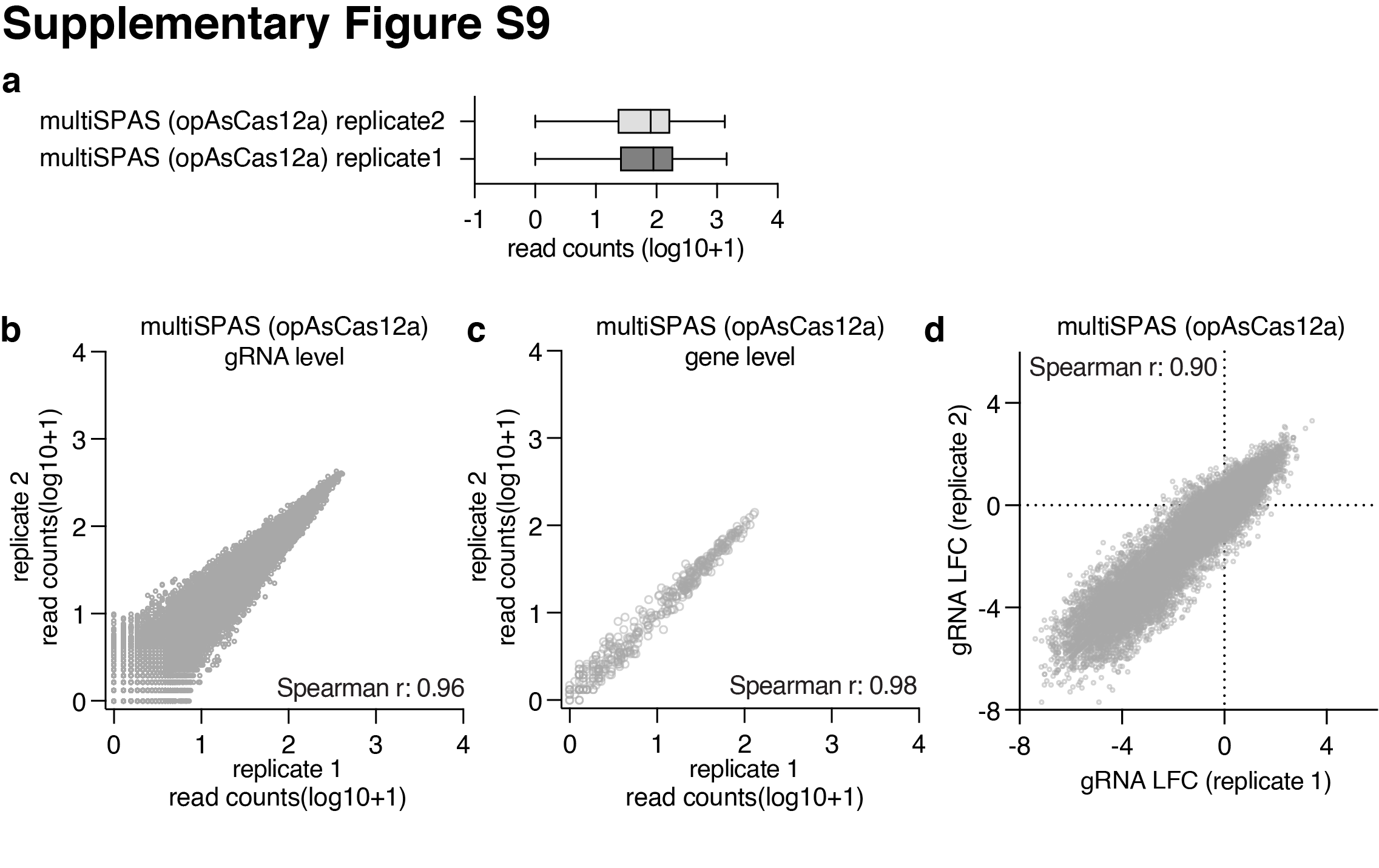

Supplement: Supplementary file 9 — Supplementary Figure S9. [file 41598_2023_34597_MOESM9_ESM.png]

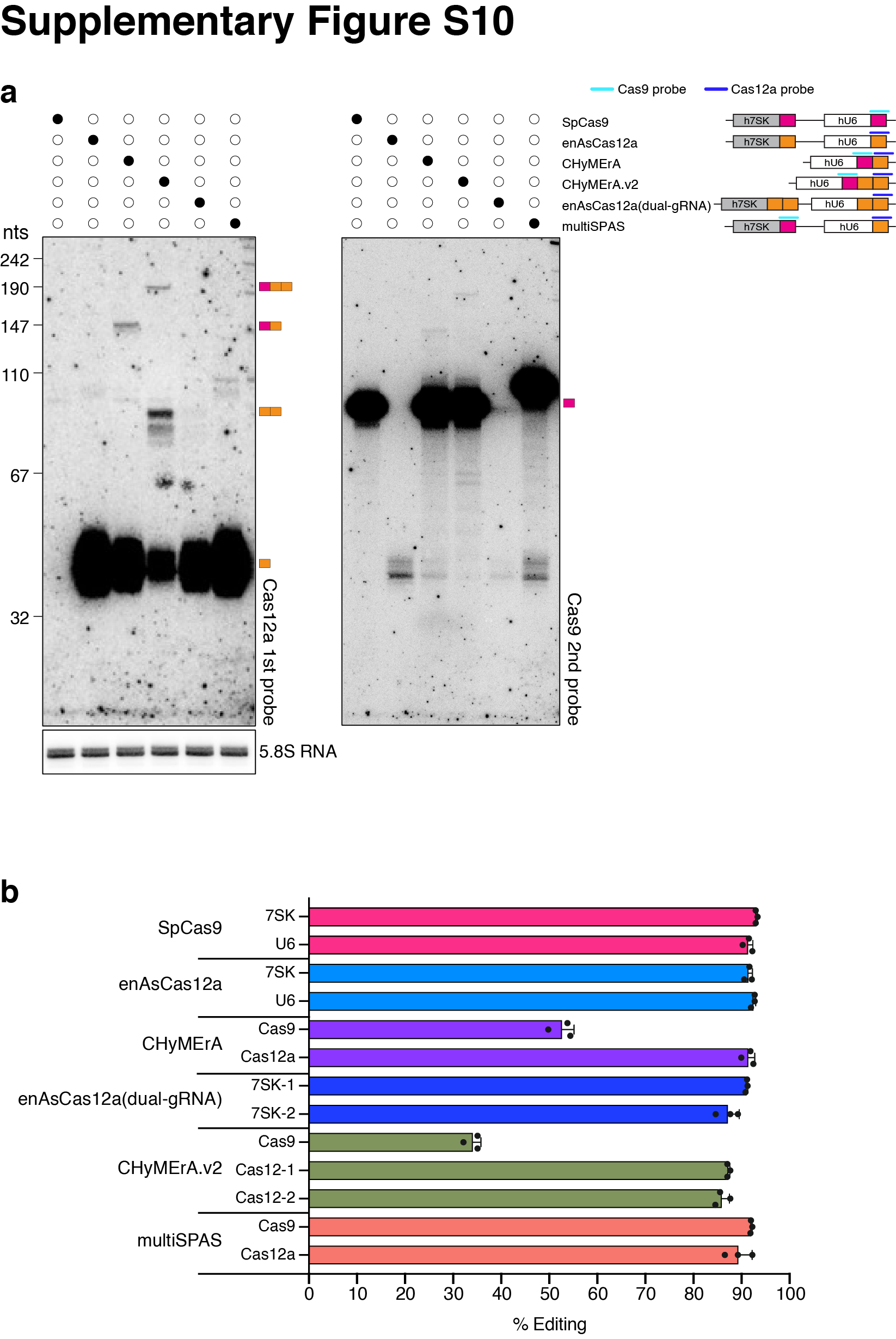

Supplement: Supplementary file 10 — Supplementary Figure S10. [file 41598_2023_34597_MOESM10_ESM.png]

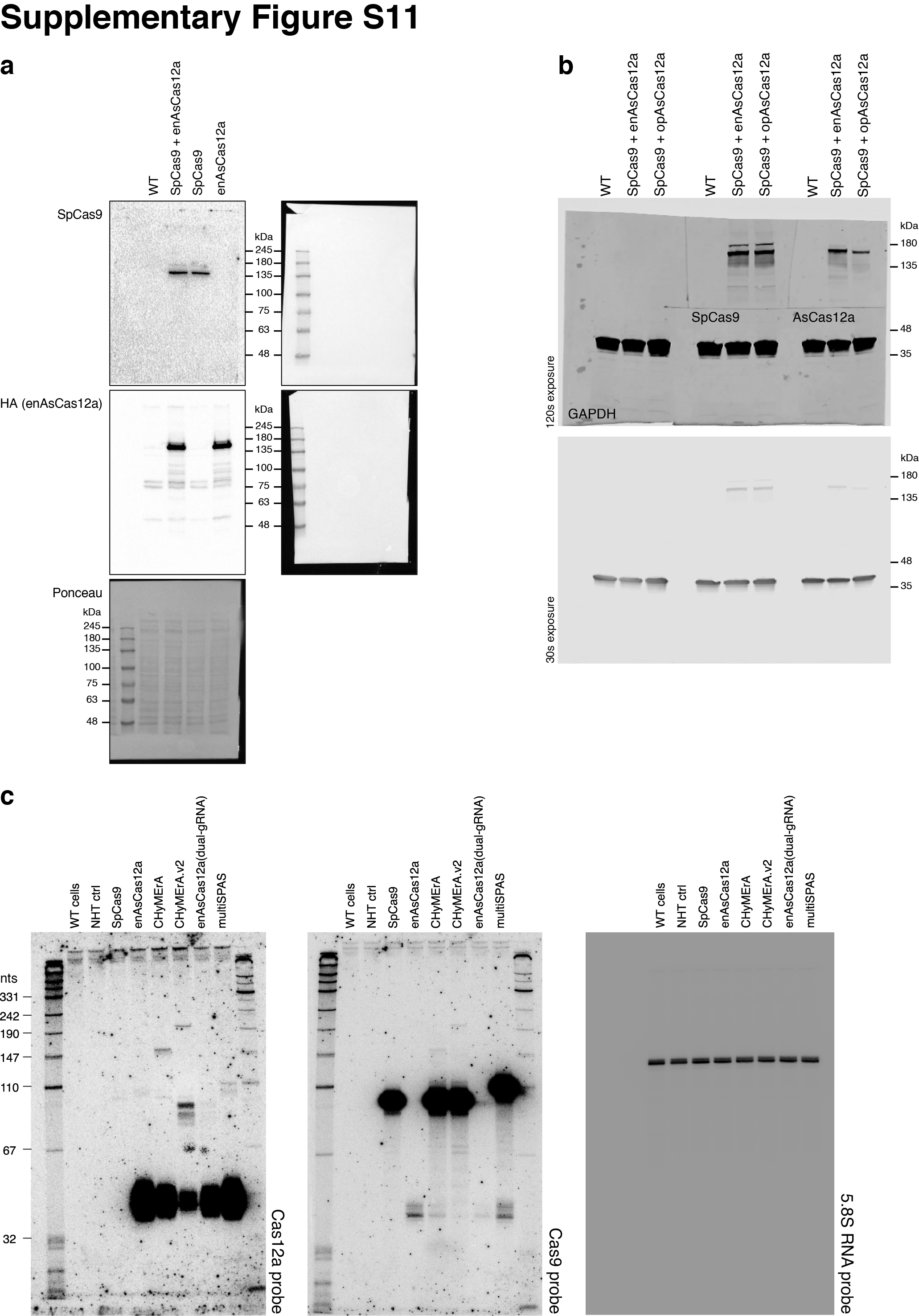

Supplement: Supplementary file 11 — Supplementary Figure S11. [file 41598_2023_34597_MOESM11_ESM.png]
