# Supplementary material for: Optimized metrics for orthogonal combinatorial CRISPR screens
Source: Sci Rep. 2023 May 6;13:7405. doi: 10.1038/s41598-023-34597-8 (PMC10164157; doi:10.1038/s41598-023-34597-8)
Supplement: Supplementary file 12 — Supplementary Legends. [file 41598_2023_34597_MOESM12_ESM.docx]

**SUPPLEMENTARY INFORMATION**

Supplementary Table S1. List of combinatorial 3Cs plasmids; Supplementary Table S2. List and sequence of gRNAs, PCR primers, northern blot probes and qPCR primers; Supplementary Table S3. List of gRNA sequences for 3Cs core essential (CE) / tumor suppressor (TS) library; Supplementary Table S4. List of DNA oligonucleotides for NGS sample preparation; Supplementary Table S5. List of total NGS read counts.

**Supplementary Figure S1. An engineered cell line model for constitutive SpCas9 and enAsCas12a expression. (a)** Immunoblot analysis of RPE1 cells constitutively expressing SpCas9 and enAsCas12a. Original blots are presented in Supplementary Fig. S11a. **(b)** Cell viability assay to assess SpCas9 and enAsCas12a activity. The cell density was measured upon DMSO and **(c)** Nutlin3 treatment after depletion of the specified genes. Data are means of replicates (n=3). Error bars represent the standard deviation of the replicates. **(d)** Receiver operating characteristic (ROC) curves of gRNAs targeting TS genes for SpCas9, enAsCas12a and CHyMErA screens. The area under the ROC curve (AU-ROC) values are given in parentheses.

**Supplementary Figure S2. Distribution and skew values of combinatorial SpCas9, enAsCas12a, and CHyMErA libraries. (a)** Cumulative distribution of SpCas9, enAsCas12a, and CHyMErA libraries respectively. Black lines represent a uniformly distributed library. The percentage indicates library representations at 90% of cumulative reads. Area under the curve (AUC) values are indicated for each library. **(b)** The histograms show the frequency of read counts and distribution skews of SpCas9, enAsCas12a, and CHyMErA libraries, respectively.

**Supplementary Figure S3. The sequencing coverage and the replicate correlations of combinatorial SpCas9, enAsCas12a, and CHyMErA libraries. (a)** ​​The boxplot visualizes the sequencing coverage for each gRNA combination. The median of the sequencing coverage is indicated. **(b)** gRNA level read count, **(c)** gene-level read count, and **(d)** gene LFC correlation of two technical replicates for SpCas9, enAsCas12a, and CHyMErA screens. Spearman’s rank correlation coefficient (r) is indicated in the plots.

**Supplementary Figure S4. SpCas9 initiates earlier cell proliferation with an effect size independent from different RNA pol III promoters. (a)** A Scatter plot to compare gRNAs derived from hU6 and h7SK RNA pol III promoters for SpCas9 and enAsCas12a. Spearman’s rank correlation coefficient r is indicated in the plots. **(b)** Cell proliferation curves for RPE1 cells expressing SpCas9 and enAsCas12a that were previously transduced with different numbers of gRNAs targeting either the AAVS1 or TP53 locus. Cells transduced with SpCas9 gRNAs are indicated in blue while enAsCas12a gRNAs are in pink. Data are means of replicates (n=3). Error bars represent the standard deviation of the replicates.

**Supplementary Figure S5. Distribution and skew values of enAsCas12a(dual-gRNA) and CHyMErA.v2 libraries. (a)** Cumulative distribution of enAsCas12a(dual-gRNA) and CHyMErA.v2 libraries respectively. Black lines represent a uniformly distributed library. The percentage indicates library representations at 90% of cumulative reads. Area under the curve (AUC) values are indicated for each library. **(b)** The histograms show the frequency of read counts and skew of the enAsCas12a(dual-gRNA) and CHyMErA.v2 libraries respectively.

**Supplementary Figure S6. The sequencing coverage and the replicate correlations of combinatorial enAsCas12a(dual-gRNA) and CHyMErA.v2 libraries. (a)** ​​The boxplot visualizes the sequencing coverage for each gRNA combination. The median of the sequencing coverage is indicated. **(b,e)** gRNA level read count, **(c,f)** gene-level read count and **(d,g)** LFC correlation of two replicate experiments for enAsCas12a(dual-gRNA) and CHyMErA.v2 screens, respectively. Spearman’s rank correlation coefficient (r) is indicated in the plots. **(h)** Receiver operating characteristic (ROC) curves of gRNAs targeting TS genes for SpCas9, enAsCas12a(dual-gRNA) and CHyMErA.v2 screens. The area under the ROC curve (AU-ROC) values are given in parentheses.

**Supplementary Figure S7. RPE1-opAsCas12a cell line and combinatorial multiSPAS library. (a)** Immunoblot analysis of RPE1 cells constitutively expressing SpCas9-enAsCas12a and SpCas9-opAsCas12a. Original blots are presented in Supplementary Fig. S11b. **(b)** qPCR for SpCas9 and AsCas12a transcripts with SpCas9-enAsCas12a and SpCas9-opAsCa12a cells. Data are means of replicates (n=4). **(c)** Cumulative distribution of multiSPAS library. The black line represents a uniformly distributed library. The percentage indicates library representation at 90% of cumulative reads. The area under the curve (AUC) value is indicated. **(d)** The histogram shows the frequency of read counts and skew of the multiSPAS library.

**Supplementary Figure S8. The sequencing coverage and the replicate correlations of combinatorial multiSPAS(enAsCas12a) library. (a)** ​​The boxplot visualizes the sequencing coverage for each gRNA combination. The median of the sequencing coverage is indicated. **(b)** gRNA level read count, **(c)** gene-level read count and **(d)** LFC correlation of two replicate experiments for multiSPAS(enAsCas12a) screen. Spearman’s rank correlation coefficient (r) is indicated in the plots. **(e)** Receiver operating characteristic (ROC) curves of gRNAs targeting TS genes for SpCas9, CHyMErA, CHyMErA.v2 and multiSPAS screens. The area under the ROC curve (AU-ROC) values are given in parentheses.

**Supplementary Figure S9. The sequencing coverage and the replicate correlations of combinatorial multiSPAS(opAsCas12a) library. (a)** ​​The boxplot visualizes the sequencing coverage for each gRNA combination. The median of the sequencing coverage is indicated. **(b)** gRNA level read count, **(c)** gene-level read count and **(d)** LFC correlation of two replicate experiments for multiSPAS(opAsCas12a) screen. Spearman’s rank correlation coefficient (r) is indicated in the plots.

**Supplementary Figure S10. Assessing gRNA processing and editing efficiency. (a)** Northern Blotting with Cas9 probe (left) and Cas12a probe (right). **(b)** gRNA editing efficiencies calculated by TIDE assay. Data are means of replicates (n=3).

**Supplementary Figure S11.** Uncropped, full-size blots of the respective cropped blots in the Supplementary Fig. S1a, S7a, and S10a.
